# Multiomics Comparison of Proline‐Rich Peptide‐Enhanced Hyaluronic Acid Gels Versus Conventional Regenerative Materials: An Early Wound‐Healing Model

**DOI:** 10.1111/jre.70032

**Published:** 2025-09-10

**Authors:** Øystein Øvrebø, Ståle Petter Lyngstadaas, Thaqif El Khassawna, Reem Jamous, Qianli Ma, Fernando Muñoz, Maria Permuy, Antonio Gonzalez Cantalapiedra, Antonio José Serrano‐Muñoz, Joana Maria Ramis, Marta Monjo, Filippo Rossi, Håvard J. Haugen

**Affiliations:** ^1^ Department of Biomaterials, Institute of Clinical Dentistry University of Oslo Oslo Norway; ^2^ Department of Chemistry, Materials and Chemical Engineering ‘Giulio Natta’ Politecnico di Milano Milan Italy; ^3^ NuPep AS Oslo Norway; ^4^ Experimental Trauma Surgery, Faculty of Medicine Justus‐Liebig University Giessen Giessen Germany; ^5^ Faculty of Pharmacy University of Jordan Amman Jordan; ^6^ Facultad de Veterinaria, Universidade de Santiago de Compostela Lugo Spain; ^7^ iBoneLab S.L Lugo Spain; ^8^ Group of Cell Therapy and Tissue Engineering, Research Institute on Health Sciences (IUNICS) University of the Balearic Islands Palma de Mallorca Spain; ^9^ Balearic Islands Health Research Institute (IdISBa) Palma de Mallorca Spain

**Keywords:** enamel‐matrix derivative, gingival regeneration, hyaluronic acid, immunomodulation, multiomics, porcine model, proline‐rich peptide, proteomics

## Abstract

**Aims:**

To compare the early wound‐healing responses to crosslinked hyaluronic acid enriched with two proline‐rich peptides (P2, P6) against unmodified hyaluronic acid and the enamel‐matrix derivative (EMD) in a porcine gingival‐detachment model.

**Methods:**

In six pigs, defects around premolars were treated with HA, HA + P2, HA + P6 or EMD. After 6 days, the sites were harvested and evaluated using histology, immunohistochemistry, multiplex cytokine assay and untargeted proteomics of the gels, which were examined, informing an integrated multiomics approach analysis.

**Results:**

Both peptide formulations improved the composite histological score compared to HA alone, and HA + P6 matched EMD in suppressing oedema, TNF‐α staining and CD80^+^ macrophages while increasing mannose‐receptor labelling. Proteomics revealed that P2 upregulated actin (Q6QAQ1) and histone H2A (F2Z5L5), consistent with active remodelling, whereas EMD reduced trypsin and trypsinogen, indicating a more stabilised wound milieu; HA + P6 showed an intermediate yet favourable profile. IL‐6 levels were lower in both peptide groups and EMD than in HA, and all gels proved noncytotoxic, with HA + P6 enhancing cell viability.

**Conclusion:**

HA + P6 suppressed TNF‐α and trypsin signalling while boosting M2‐linked markers and proresolution proteins to the same extent as EMD, whereas plain HA did not. Thus, adding a short proline‐rich peptide confers EMD‐like early immunomodulation on a fully synthetic HA carrier. These observations, although limited to 6‐day soft tissue healing, warrant longer studies in true periodontal regeneration models.

## Graphical abstract

A proline‐rich peptide (P6) embedded in crosslinked hyaluronic acid replicates EMD's early anti‐inflammatory and proremodelling signature in a porcine gingival model, highlighting a fully synthetic, ethically sound candidate for periodontal regeneration.

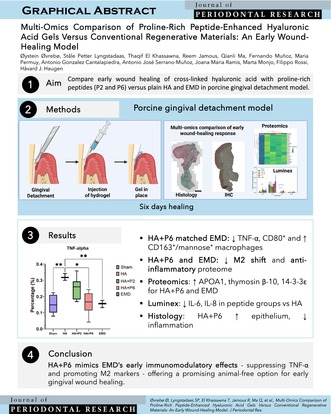

**Summary**

- Background
  ○ Enamel‐matrix derivative remains a well‐established benchmark among methods for periodontal regeneration; however, its animal origin entails cost, supply and ethical constraints. In contrast, newer crosslinked hyaluronic acid delivers variable clinical benefits. There is a need for a purely synthetic alternative that can replicate the biological advantages of EMD.
- Added value of this study
  ○ In a multiomics porcine gingival‐detachment model, crosslinked hyaluronic acid augmented with two short proline‐rich peptides (P2, P6) reproduced the early immunomodulatory signature of EMD. The lead formulation (HA + P6) suppressed TNF‐α, promoted M2‐oriented macrophages and restored key proresolution proteins, achieving the same histological score as EMD while remaining fully synthetic.
- Clinical implications
  ○ A synthetic peptide‐enhanced HA gel may offer clinicians an injectable, off‐the‐shelf alternative to EMD for gingival reattachment and mucogingival surgery, thereby eliminating the need for animal‐derived ingredients. Longer follow‐ups in RCTs are necessary before clinical recommendations for periodontal regeneration can be made.

## 1 Introduction

The periodontal tissue surrounds the teeth and is essential for their structural stability. However, the tissue is prone to degradation through periodontal disease, which affects more than 70% of the population above 65 years of age [1]. It is characterised by the formation of a periodontal pocket, with the gingival sulcus deepening due to apical migration of the junctional epithelium and detachment of connective tissue from the root surface [2]. The pocket commonly harbours bacterial plaque and subgingival calculus, exacerbating tissue degradation. To prevent the progression of periodontitis, it is therefore beneficial to control inflammation by removing plaque, calculus and biofilm from the affected area and stimulating the reattachment of the gingiva.

In a recent consensus report for clinical practice guidelines from the European Federation of Periodontology, it was recommended that for early‐stage (Stage I–II) to late‐stage (Stage III–IV) periodontitis, the first step should focus on controlling the local and systemic risk factors. A second step involving nonsurgical instrumentation should then be performed to remove the subgingival biofilm [3]. A systematic review by Suvan et al. confirmed that mechanical instrumentation is an effective measure for infection control [4]. However, there are still about 30% of the cases where mechanical instrumentation is insufficient to reach the endpoints of therapy [5, 6].

For these cases refractory to nonsurgical therapy, a third treatment step is required. In such cases, surgical intervention is recommended for PPD ≥ 6 mm [3]. When intrabony defects or Class II furcation defects are present, treatment with regenerative materials, such as enamel‐matrix derivative (EMD), is recommended [3]. Clinically, EMD is dissolved in a sterile gel carrier of polypropylene glycol alginate, where the EMD mixture consists primarily of amelogenin (90%) [7]. The clinical literature on this product encompasses the treatment of intrabony defects and root coverage procedures [8, 9, 10, 11]. EMD therapeutic effects are mediated by the regulation of dentin sialoprotein, TGF‐β, collagen and bone morphogenetic proteins (BMPs) [7]. Crosslinked hyaluronic acid has also been used in periodontal regeneration and has been demonstrated to be effective in treating both intrabony defects and gingival recession successfully [8, 9]. However, Pilloni et al. showed that although both products provide clinically meaningful regeneration, the reduction in PPD was significantly greater for EMD at all time points (12, 18 and 24 months) and in clinical attachment level (CAL) at 18 and 24 months [10]. To determine what drives this difference in response, it is essential to understand the biological activity of the key ingredients: EMD (30 mg/mL) and hyaluronic acid crosslinked with 1,4‐butanediol diglycidyl ether (HA‐BDDE—16 + 2 mg/mL NaHA). Additionally, the EMD is animal‐derived, and strict sourcing requirements are in place to minimise the risk of pathogen transmission, including viruses, bacteria and prions, as outlined in ISO 22442‐1 [11, 12]. This makes the sourcing and preparation of animal‐derived proteins expensive. Moreover, there are ethical concerns related to the animal origin [13]. Therefore, there is a need for novel bioactive molecules of synthetic origin that can improve the biological response to medical devices [14].

EMD was introduced to the clinic in the late 1990s [15]. Since then, crosslinked hyaluronic acid has been introduced as an alternative. However, the performance remains inferior [10], illustrating the need for fully synthetic solutions with improved bioactivity. A series of proline‐rich peptides that mimic EMD and have demonstrated similar bioactivity in vitro and in vivo [16, 17, 18] have been designed. Among these, candidates P2 and P6 (Table 1) have demonstrated high potency. It was hypothesised that these peptides could be incorporated into hyaluronic acid gels to enhance the bioactivity in a novel product for periodontal regeneration, a novel application for these peptides.

**TABLE 1:** Peptide sequences of the two peptide variants; H, histidine; L, leucine; M, methionine; P, proline; Q, glutamine; S, serine; V, valine—Covered by the patent: [19].

Peptide	Peptide sequences
P2	PLV PSQ PLV PSQ PLV PSQ PQ PPLPP
P6	PHQ PMQ PQP PVH PMQ PLP PQ PPLPP

In this research, it was hypothesised that a synthetic peptide‐enhanced hyaluronic acid gel could mimic EMD immunomodulatory effects in the early wound‐healing acute porcine gingival‐detachment model. The secondary objective was to understand the local biological response of the suggested solution compared to clinically used biomaterials, EMD and crosslinked HA. This model isolates the immediate epithelial–connective interface and macrophage polarisation events common to all flap procedures, while deliberately excluding structural bone healing. The response was analysed using histology to evaluate inflammatory and morphological changes to the tissue. Multiomics was employed to investigate protein expression, identify specific proteins involved in the response, and perform integrated statistical analysis.

## 2 Methods

### 2.1 Test Groups

The hyaluronic acid (HA) was crosslinked with 1,4‐Butanediol diglycidyl ether (BDDE) and mixed with 10% sodium hyaluronate in a formulation comparable to that of HyaDENT BG.

Emdogain (EMD) was acquired from Institut Straumann AG, Basel, Switzerland. HA with 50 μg/mL P2 (HA + P2) and with 50 μg/mL P6 (HA + P6) was manufactured in accordance with ISO 13485:2016. The peptide concentration was based on the highest bioactivity displayed in former studies [16, 17, 18, 20, 21, 22, 23]. The peptide sequences of P2 and P6 are shown in Table 1.

### 2.2 Animal Handling

A total of seven conventional pigs (Breeding farm, Spain) (age: 2 months, weight: 18.750–15.135 kg, quarantine period: 8 days) were used for this study. All experiments were conducted in accordance with national legislation and community guidelines, following the authorisation of the competent, autonomous authority at the facilities available to the Rof Codina Foundation (CeBioVet facility, Lugo, Spain) for this purpose (Approval number 02/20/LU001 from the Galician Government). The animals were kept as a group identified by subcutaneous microchips. They were housed in an area with natural light, fresh air and a regulated temperature. The animals were fed a conventional granulated diet for their species and had access to a water supply. During the study, they were visited daily by people trained in laboratory animal science.

All procedures were performed using general anaesthesia. The animals were premedicated with an intramuscular combination of medetomidine 20 μg/kg (Sededorm; Vetpharma Animal Health S.L., Spain), ketamine 10 mg/kg (Ketamidor, Karizoo Laboratories S.A., Spain), midazolam 0.3 mg/kg (Midazolam Normon, Normon Laboratories S.A., Spain) and morphine 0.3 mg/kg (Morfina Serra, Serra Pamies Laboratories S.A., Spain) for sedation and pain control under veterinary care. Furthermore, an intravenous injection of meloxicam 0.2 mg/kg (Metacam, Boehringer Ingelheim S.A., Spain) was administered to provide adequate analgesia. General anaesthesia was induced with the intravenous administration of propofol (2–4 mg/kg; Propofol Lipuro, B. Braun VetCare S.A., Spain) and maintained with isoflurane (Vetflurane, VIRBAC Laboratories S.A., Spain), an inhalational anaesthetic agent. During anaesthesia, the animals were monitored via electrocardiography, capnography, pulse oximetry and noninvasive blood pressure. Antibiotic prophylaxis was administered before surgery using cefazoline at a dose of 22 mg/kg (Cefazolina Normon, Normon Laboratories S.A., Spain). After surgery, one dose of amoxicillin trihydrate (Amoxil retard, SYVA Laboratories S.A., Spain) at 15 mg/kg was administered to the pigs for 48 h of antibiotic coverage. The animals were monitored daily and during the interventions by a veterinarian accredited and trained in the science of laboratory animals (Categories B or C, Functions a, b and c).

### 2.3 Surgery and Sample Extraction

The gingival tissue of three premolars on each side of both the upper and lower jaws was surgically detached from the teeth using a scalpel. Any residual periodontal ligament was gently removed with a spatula. Each gingival flap measured approximately 4 × 3 mm, with a depth that reached the periosteum and a split–thickness extension to the mucogingival junction. The inner epithelial lining of the gingival flaps was excised using a scalpel to ensure a consistent wound bed. Haemostasis was achieved prior to the application of the experimental gels. There were no postoperative oral hygiene protocols applied during the 6‐day experiment.

Each quadrant of the oral cavity was randomly assigned to one of the treatment groups. Sham, HA + P2, and HA + P6 treatments were applied in all six animals. HA and EMDOGAIN (commercially available EMD) were applied to three animals each. In the seventh animal, only EMD and HA treatments were used, with two application sites per treatment group assigned to the lower quadrants and one per group to the upper quadrants.

After 6 days, the animals were euthanised following sedation with medetomidine (20 μg/kg, Sededorm; Vetpharma Animal Health S.L., Spain), ketamine (10 mg/kg, Ketamidor; Karizoo Laboratories S.A., Spain) and midazolam (0.3 mg/kg, Normon; Normon Laboratories S.A., Spain). Anaesthesia was induced with propofol (2–4 mg/kg, Propofol Lipuro; B. Braun VetCare S.A., Spain) and followed by euthanasia via intravenous overdose of pentobarbital (200 mg/kg, Dolethal; Vetoquinol E.V.S.A., Spain). The gingival tissue samples from the center third of the defects were explanted, dissected and fixed in 70% ethanol, then transferred to 4% paraformaldehyde (PFA) for 24 h.

### 2.4 Histology and Immunohistochemical Preparation and Histomorphological Analysis

After euthanasia, gingival tissues were harvested and fixed in phosphate‐buffered 4% paraformaldehyde (PFA) (Cat. no. 158127, Sigma‐Aldrich, St. Louis, MO, USA) at 4°C for 24 h. Samples were subsequently embedded in Technovit 9100 NEW (Heraeus Kulzer, Hanau, Germany) following the manufacturer's protocol. Sectioning was performed using a rotary microtome (HM 355S, Thermo Fisher Scientific, Karlsruhe, Germany) at a thickness of 5 to 6 μm Kawamoto's film method (SECTION‐LAB Co. Ltd., Hiroshima, Japan) was applied during sectioning to maintain tissue integrity and minimise the detachment of biomaterial from the sections.

Histological evaluation was conducted on toluidine blue and Masson‐Goldner trichrome‐stained sections to assess overall tissue morphology and inflammatory status. Inflammation grading was based on epithelial integrity, oedema and inflammatory infiltration using a three‐tier scale: 3 (normal, keratinisation maintained, noninflamed), 2 (moderately altered) and 1 (compromised or infected) (Table 2). Two independent investigators scored the slides, one of whom was blinded to the treatment groups. Substantial inter‐rater reliability was confirmed using Cohen's kappa (*κ* = 0.72). For immunohistochemical analysis, sections were first treated with 3% hydrogen peroxide solution (Merck KGaA, Darmstadt, Germany) for 5 min to quench endogenous peroxidase activity. After rinsing in phosphate‐buffered saline (PBS), nonspecific binding was blocked using a commercial protein blocking solution (DAKO, X0909, Agilent Technologies, Santa Clara, CA, USA). Sections were then incubated overnight at 4°C with primary antibodies: anti‐CD80 (ab64116, Abcam, Cambridge, UK), anti‐CD163 (ab182422, Abcam), anti‐CD206 (ab64693, Abcam), anti‐IL‐10 (ab34843, Abcam) and anti‐TNF‐α (ab6671, Abcam), all diluted in DAKO antibody diluent (S0809, Agilent Technologies).

**TABLE 2:** Grading scale for only the histology samples. The sample score was the average of the three scorings: Epithelium, oedema and inflammatory infiltrate.

Grading scale	Epithelium	Oedema	Inflammatory infiltrate	General description/comments
3—Normal	Strong keratinisation maintained	None	None	Healthy tissue without signs of inflammation or damage
2—Moderate	Slightly irregular or thin	Mild oedema	Moderate	There is some evidence of inflammation and oedema, but the structure is still intact
1—Abnormal	Weakened, breached	Significant oedema	Extensive infiltration/infection	Active infection and significant damage; tissue may be necrotic

Following primary antibody incubation, the slides were treated with biotinylated goat antirabbit IgG secondary antibody (BA‐1000, Vector Laboratories, Burlingame, CA, USA) and then processed using the VECTASTAIN Elite ABC Kit (PK‐6100, Vector Laboratories) for signal amplification. Immunoreactivity was visualised using the NovaRED Peroxidase Substrate Kit (SK‐4800, Vector Laboratories) and counterstained with haematoxylin (Cat. no. 6765009, Thermo Fisher Scientific, Waltham, MA, USA). Slides were mounted using Vitro‐Clud mounting medium (R. Langenbrinck GmbH, Emmendingen, Germany) and imaged using a Leica DM5500 microscope (Leica Microsystems GmbH, Wetzlar, Germany), then analysed in ImageJ as earlier described [24].

### 2.5 Protein Extraction

After RNA extraction (samples degraded; see the limitations section), the remaining interphase and organic phases from TRIzol Reagent (ThermoFisher Scientific, Madrid, Spain) were subjected to protein extraction, following the manufacturer's guidelines. Briefly, 0.3 mL of 100% ethanol (Labkem, Barcelona, Spain) was added to each sample for every 1 mL of TRIzol. The tubes were centrifuged, and the DNA pellets were stored at −20°C. Supernatants containing proteins were subjected to dialysis using Slide‐A‐Lyzer dialysis cassettes (2000 MWCO, Thermo Fisher Scientific, Madrid, Spain). Dialysis was performed against three changes of 0.1% SDS at 4°C, with the first change made after 16 h, the second after 4 h and the last after an additional 2 h. The protein solution was centrifuged for 10 min at 10 000 × *g* at 4°C before transferring the supernatant into a new container. To enhance protein recovery, the remaining protein pellets were solubilised in 100 μL of a buffer containing Tris–HCl 0.05 M (PanReac AppliChem, Monza, Italy), urea 4 M (PanReac, Barcelona, Spain) and SDS 0.05% (Sigma‐Aldrich, Darmstadt, Germany) at a pH of 8, as described by Hummon et al. [25]. If necessary, heat (50°C) was applied to the samples that remained insoluble after the addition of the buffer. The samples were then stored at −20°C for further processing.

### 2.6 Protein Quantification and Luminex

After solubilisation, protein concentration was measured using the Pierce BCA Protein Assay Kit (Thermo Scientific, Rockford, USA), following the manufacturer's instructions. In short, samples were diluted 25‐fold and loaded into a 96‐well ELISA plate. Two different calibration curves were prepared for each protein fraction, supernatant and solubilised pellet, using 0.1% SDS as the diluent for the supernatant and buffer (Tris–HCl 0.05 M, urea 4 M, and SDS 0.05%) for the solubilised pellet, respectively. Absorbance was read at 562 nm using a PowerWave HT plate spectrophotometer (Biotek). Luminex analysis was conducted using the commercial preconfigured immunoassay multiplex assay MILLIPLEX Porcine Cytokine/Chemokine Magnetic Bead Panel (Merck, Darmstadt, Germany). The supernatants obtained as described above were processed according to the manufacturer's protocols (Porcine Cytokine/Chemokine Magnetic Bead Panel Catalogue). The following 13 cytokines were analysed: GM‐CSF, IFNγ, IL‐1α, IL‐1β, IL‐1Ra, IL‐2, IL‐4, IL‐6, IL‐8 (CXCL8), IL‐10, IL‐12, IL‐18, TNF‐α. Multianalyte profiling was performed using the Luminex200 system (Luminex Corporation, Austin, TX, USA) after overnight incubation and acquired fluorescence data were analysed by the xPONENT3.1 software.

### 2.7 Mass Spectrometry and Proteomics

The digested samples were analysed in a nanoElute system coupled to a timsTOF pro (Bruker). The peptides were separated by liquid chromatography in an Aurora Elite column (C18, 1.5 μm beads, 75 μm inner diameter, 15 cm length; IonOptics) using a flow rate of 200 nL/min with 0.1% formic acid (solvent A) and 0.1% formic acid in acetonitrile (solvent B). A 60‐min gradient was used, starting from 2% solvent B to 35% at a column temperature of 50°C. The mass spectrometer was operated in data‐dependent mode to isolate automatically and fragment multiple charged precursors (top 10). The data obtained from the MS were processed and quantified using label‐free quantification methods. To ensure comparability across samples, the raw protein quantification data were normalised (Figure S1). The normalisation was performed using the ‘equalMedianNormalizations’ function from the ‘DEqMS’ package in the statistical software R, which adjusts the data to account for systematic differences across samples. The log2 transformation was applied to stabilise variance and make the data distribution more normal‐like. These data identified the 40 most expressed proteins by evaluating median absolute deviation (MAD) in the Heatmap visualisation. Furthermore, principal component analysis (PCA—‘factoextra’ package) and partial least‐squares discriminant analysis (PLS‐DA—‘mixOmics’ package), freely available on Bioconductor (https://bioconductor.org) were applied. Lastly, differential expression analysis (DEA) was carried out using the ‘limma’ package [26]. This method identified differentially expressed proteins (DEPs) by fitting a linear model to the expression data and conducting empirical Bayes moderation of the standard errors. DEPs were identified based on a log2(|Fold Change (FC)|) threshold of 0.585 and a *p* value threshold of 0.05. RStudio, Version: 2024.04.2 + 764, was used for plot visualisation.

### 2.8 Multiblock Confirmatory Model

To confirm whether the same covariation structure persisted after dimension reduction, the four data blocks—Histology, IHC, Luminex and Proteome—were integrated using multiblock partial least‐squares discriminant analysis (block PLS‐DA) with the DIABLO algorithm implemented in mixOmics (v6.24). An empirical design matrix with an off‐diagonal weight of 0.1 linked the blocks while preventing overfitting. Two latent components were extracted—the minimum number that yielded stable classification accuracy in preliminary tuning. Model performance was evaluated using a leave‐one‐pig‐out cross‐validation procedure repeated 10 times; the average overall accuracy, balanced error rate and area under the ROC curve were recorded. Sample projections (scores) were plotted on the LV1–LV2 plane, and 95% confidence ellipses were generated for each treatment group. Only variables retained by the DIABLO feature‐selection procedure (keepX = 25 per block) were interpreted in the text. Group centroids and 95% confidence ellipses were plotted from the resulting score matrix (*χ* ^2^ distribution, 2 d.f., *p* = 0.05; matplotlib 3.8). To test the robustness of class separation, we computed the ratio of between‐ to within‐cluster sums of squares (SSᵦ/SS𝑤) for the observed labels and 1000 random relabellings; the empirical *p* value equals the proportion of permuted ratios at least as large as the observed one. Finally, the early profiles of HA + P6 and EMD were compared variable‐by‐variable with Welch's *t*‐test, and Benjamini adjusted the resulting *p* values with the Hochberg procedure, controlling the false discovery rate at *q* < 0.05. All analyses were carried out in R (version 4 3·) based on scripts by Zhang and Datta [27].

### 2.9 In Vitro Biocompatibility Testing

The biocompatibility of the gels was assessed using in vitro cell viability testing with a CCK8 Assay, following the method outlined in ISO 10993‐5 [28]. The technique has been described previously [29]. In brief, a 24‐well plate was seeded with 40 000 mouse preosteoblastic cells (MC3T3‐E1) in each well and 1 mL of cell medium. The gel was introduced using cell inserts with a 0.4 μm polyethene terephthalate (PET) membrane. EMD was excluded from this analysis due to its limited availability, and its biocompatibility has been primarily established clinically.

### 2.10 Statistics

Data distribution was first assessed for normality using the Kolmogorov–Smirnov test, followed by a Holm–Sidak method for normality verification, presenting data as means with standard deviation if normally distributed and median with interquartile range if not. Both one‐way and two‐way ANOVA and Tukey's post hoc tests were used for comparing parametric datasets. The Mann–Whitney test was used for the nonparametric dataset. All data obtained were analysed using GraphPad Prism version 10.1 (GraphPad Software Inc., CA, USA). Spearman's bivariate correlation was used to analyse the correlation plots. The results were interpreted as follows: no correlation if |*r*| < 0.2; correlation if 0.2 < |*r*| < 0.5; and strong correlation if 0.5 < |*r*| < 1. A negative r indicated a negative correlation, whereas a positive r indicated a positive correlation. All graphical representations were performed on GraphPad Prism and Biorender. A priori sample size estimation was conducted using G*Power version 3.1 [30], informed by effect sizes reported in previous comparable studies. To achieve a statistical power (1–*β*) of 0.85 and a Type I error rate (*α*) of 0.05, a minimum of six samples per experimental group was calculated to be necessary. Statistical significance was defined as a *p* value of less than 0.05 for all analyses. The study design and reporting adhere to the ARRIVE 2.0 guidelines for preclinical animal research as recommended by the EQUATOR Network.

## 3 Results

All six animals appeared healthy during the 6‐day follow‐up period, with no signs of systemic or local oral reactions or toxicity from the procedure or treatment. However, one animal was lost due to causes unrelated to the biomaterials used (during the peri‐anaesthetic period, due to a bradyarrhythmic cardiac event), resulting in an uneven number of defects per group.

### 3.1 Histology and Histomorphometry

Masson‐Goldner Trichrome staining was employed to obtain a detailed description of the gingival tissue's morphology. While the stain is primarily intended for connective tissue coloration, it was discovered that it is also helpful in detecting alterations in the epithelium, identifying oedema and assessing inflammatory infiltrates and acute local infections. Each feature was assigned a score ranging from 1 to 3, corresponding to compromised to normal physiology, and then the scores were averaged. The scores were then averaged to determine an overall morphology score for each sample.

**FIGURE 1:**
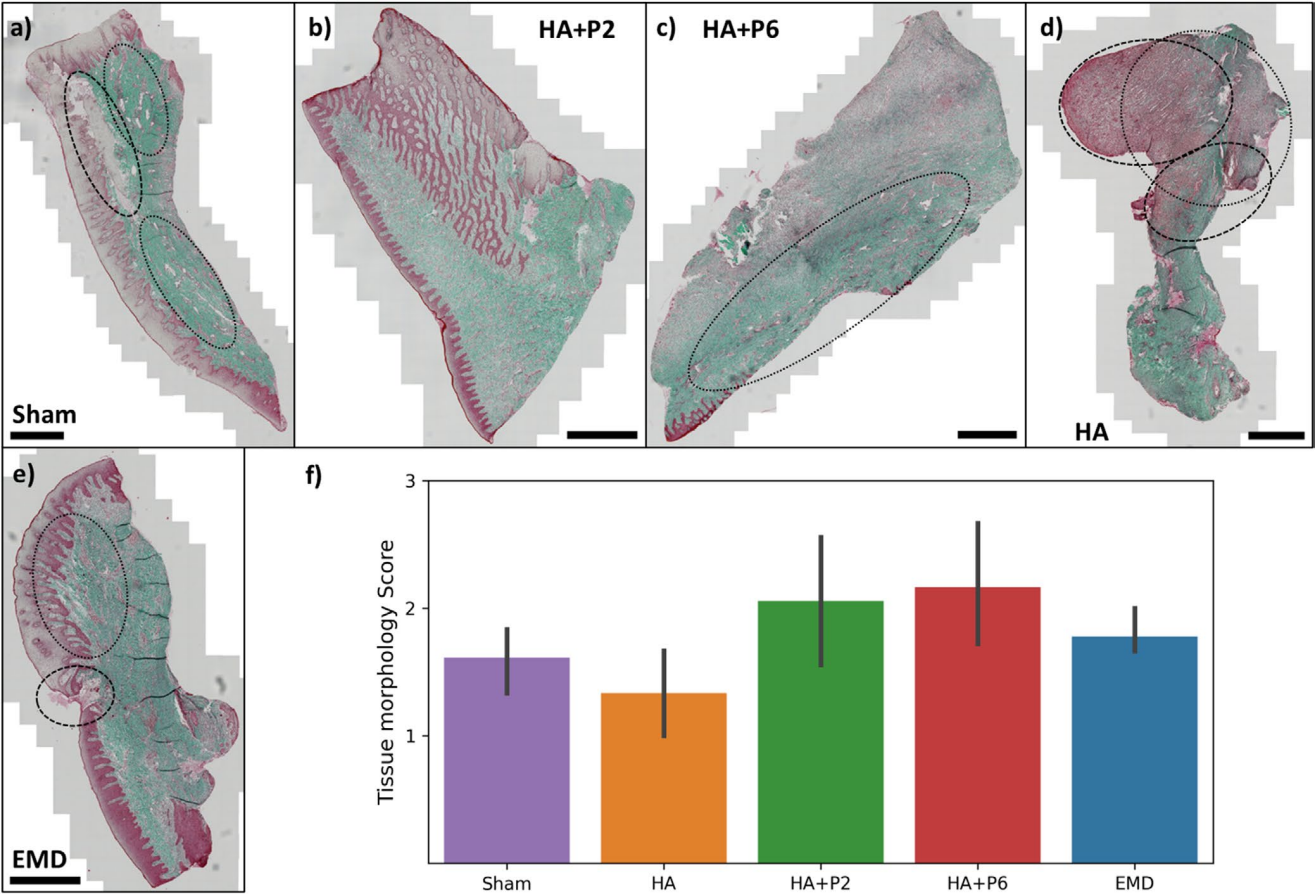
Representative Masson‐Goldner trichrome stain slides with dashed line circling in regions of active inflammation (Sham: Necrotic/encapsulated tissue; HA: Inflammatory infiltrate; EMD: Active infection) and dotted line illustrating oedema (a–e). All histology images, except the EMD (pig 6), are from pig 2. Scale bar = 1 mm. The graph displays the mean tissue morphology score (f) for each group, where 1 indicates compromised tissue morphology, and 3 indicates normal tissue morphology. The score of each sample was an average of the three indicators of epithelial physiology, oedema and inflammatory infiltrate/active infection. *n* = 6 defects for sham, HA + P2, HA + P6, and *n* = 3 defects for HA and EMD.

Figure 1 presents representative histology slides for each group. The scoring table (Table S1) and all histology images are included in Figures S5–S10, along with digital images of the animals. Overall, introducing peptides HA + P2 and HA + P6 demonstrated the most favourable results, with controlled inflammation and robust tissue healing, while HA consistently produced the poorest outcomes, marked by severe inflammation and tissue damage. Regarding the epithelium, HA + P2 and HA + P6 maintained strong, keratinised tissue with minimal irregularities. Sham and EMD showed variable results, with some samples exhibiting thinning or breaches. In contrast, HA consistently displayed weak, thin and frequently breached epithelium. HA + P2 and HA + P6 maintained moderate levels of oedema that did not impede healing. Sham and EMD exhibited variable oedema, often associated with inflammation, while HA consistently presented severe oedema, frequently linked to poor healing and active infections. Regarding inflammatory infiltration, HA + P2 and HA + P6 effectively managed inflammation, with moderate infiltrate supporting tissue remodelling. Sham and EMD showed mild to moderate infiltrate, with signs of ongoing inflammation in some cases. HA displayed intense infiltration, often accompanied by active infections, necrotic tissue and poor healing outcomes. This is reflected in the mean tissue morphology score displayed in Figure 1f.

**FIGURE 2:**
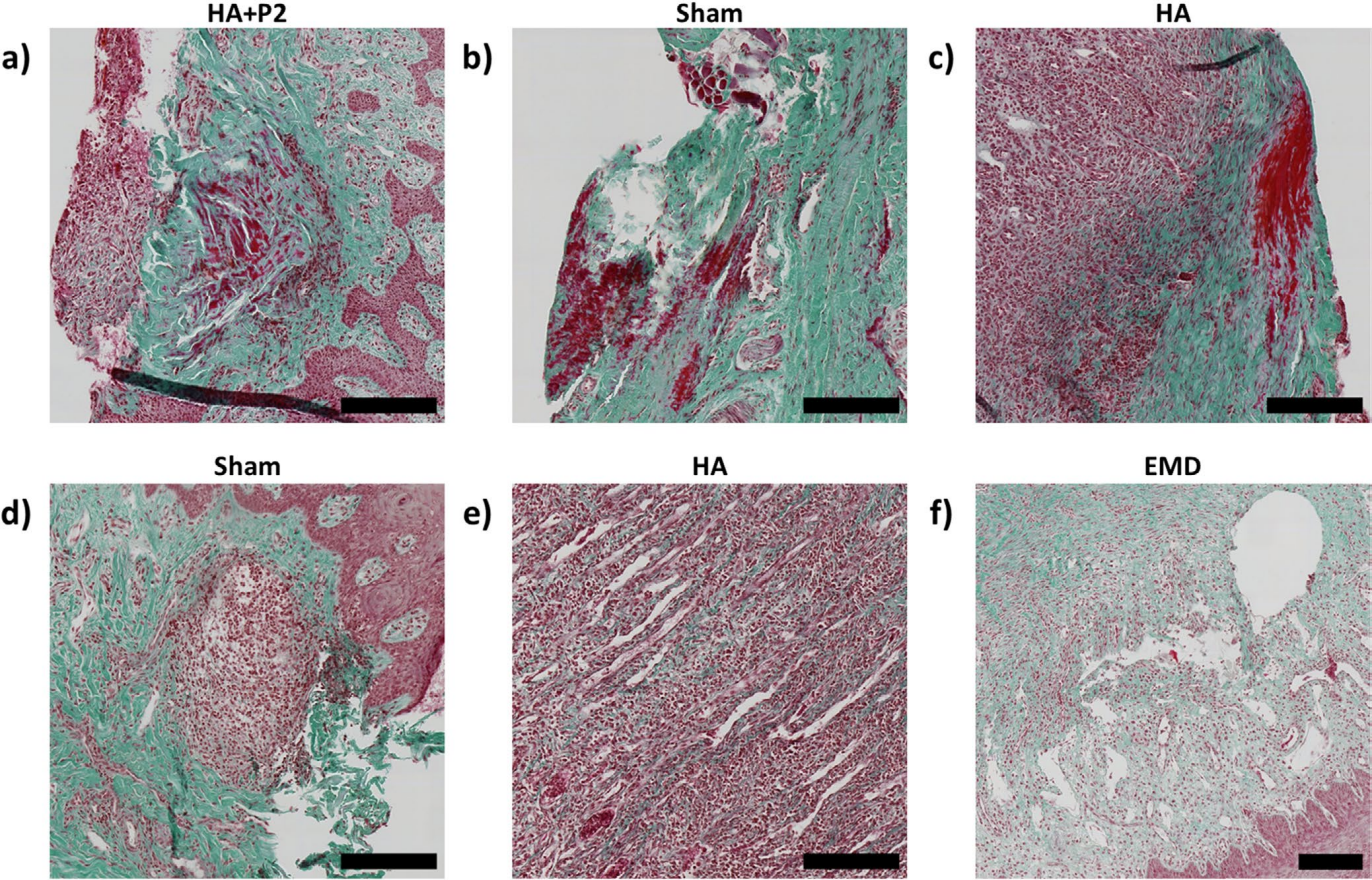
Zoom in on histology slide features–coagulation (a–c), inflammation (c–e) and oedema (e, f). Scale bar: 200 μm.

More detailed examples of the histological findings are displayed in Figure 2. The coagulum was observed in Figure 2a–c. A significant difference was that inflammatory cells circled the coagulum for the HA + P2 sample, suggesting active remodelling. This was not observed for the HA or sham group. In Figure 2c–e, regions with inflammatory infiltration were observed, particularly for the HA groups, while extensive oedema was observed in Figure 2e,f. Toluidine blue stains are displayed in Figures S11–S16.

### 3.2 Immunohistochemistry and Histomorphometry

**FIGURE 3:**
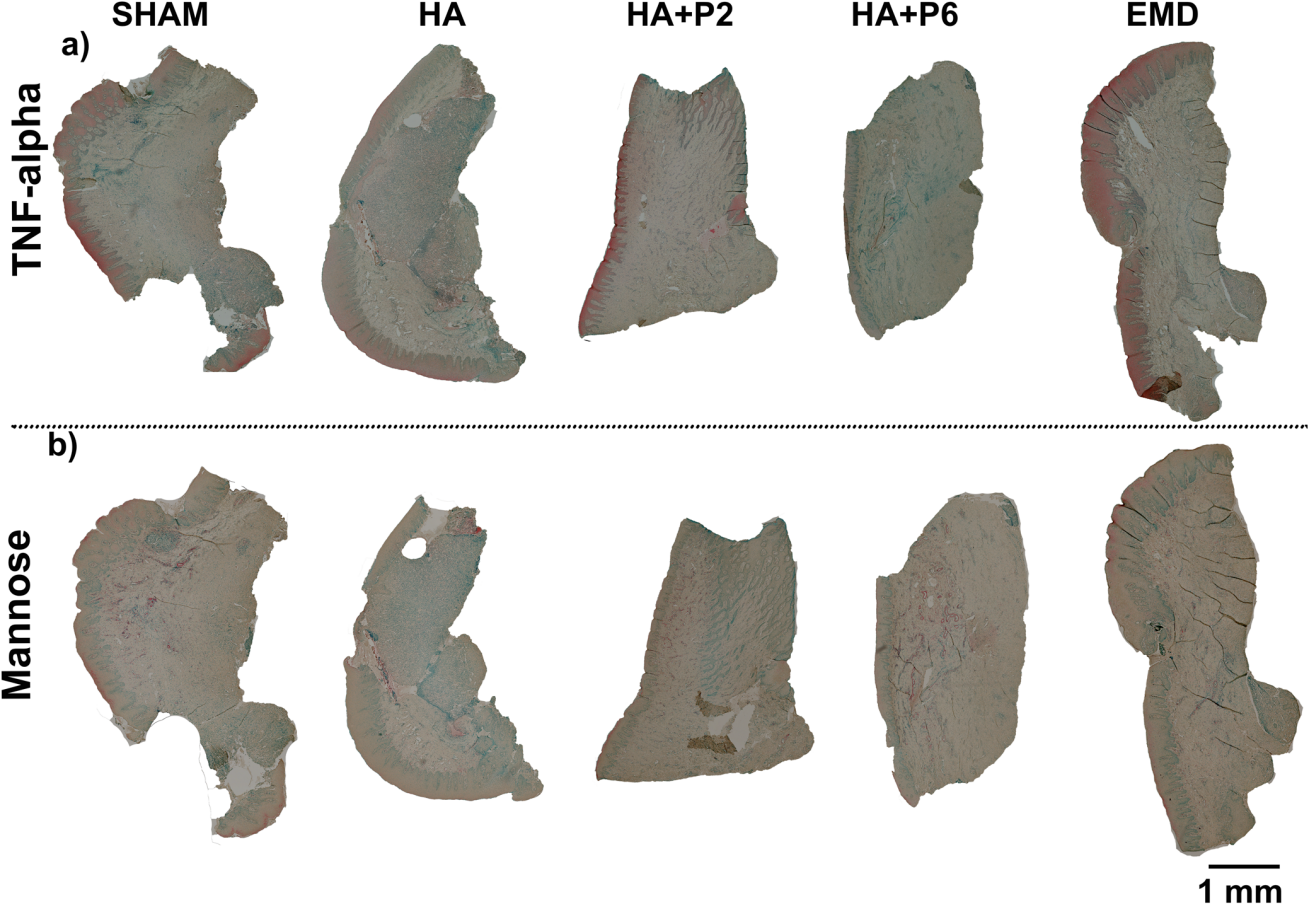
Selected representative Immunohistochemistry staining of TNF‐alpha (a) and Mannose (b).

Representative sections stained for the proinflammatory cytokine TNF‐α (panel a) and for mannose receptor (panel b) reveal treatment‐dependent modulation of the early inflammatory micro‐environment (Figure 3). Sham defects exhibited a diffuse, low‐to‐moderate TNF‐α signal and weak mannose‐positive labelling, representing the baseline innate response to an ungrafted cavity. HA implantation markedly intensified TNF‐α immunoreactivity throughout the defect, whereas mannose staining remained faint, denoting a sustained M1‐skewed inflammatory profile elicited by the hyaluronic acid alone. HA + P2 reduced the overall TNF‐α burden compared with unmodified HA, while producing a marginal increase in mannose‐positive cells, indicating partial attenuation of the proinflammatory milieu. HA + P6 further suppressed TNF‐α expression to near‐background levels. It elicited the most homogeneous mannose‐receptor staining among the experimental groups, consistent with a pronounced shift toward an anti‐inflammatory, M2‐like phenotype. EMD mirrored the HA + P6 pattern, displaying minimal TNF‐α and prominent mannose labelling, corroborating its known capacity to foster a prohealing macrophage response. Collectively, peptide P6 and EMD most effectively dampened TNF‐α–driven inflammation while promoting mannose‐expressing macrophages, whereas unmodified HA sustained a robust proinflammatory state (Figure 3a,b).

**FIGURE 4:**
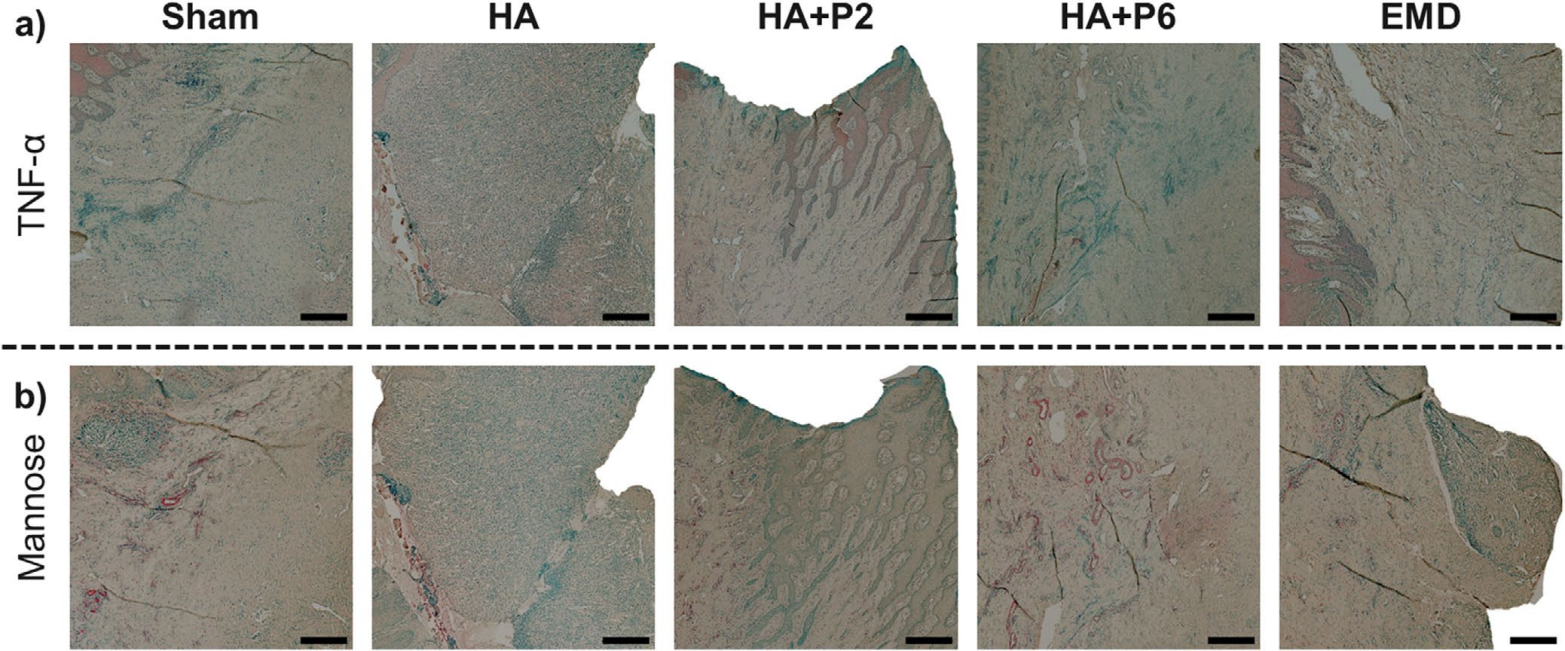
Zoom‐in of selected representative immunohistochemistry staining of TNF‐alpha (a) and mannose (b). Scale bar = 250 μm.

Representative immunohistochemical micrographs (scale bar = 1 mm) illustrate distinct immune profiles among the five experimental conditions. Further zoom‐in of representative areas is presented in Figure 4.

**FIGURE 5:**
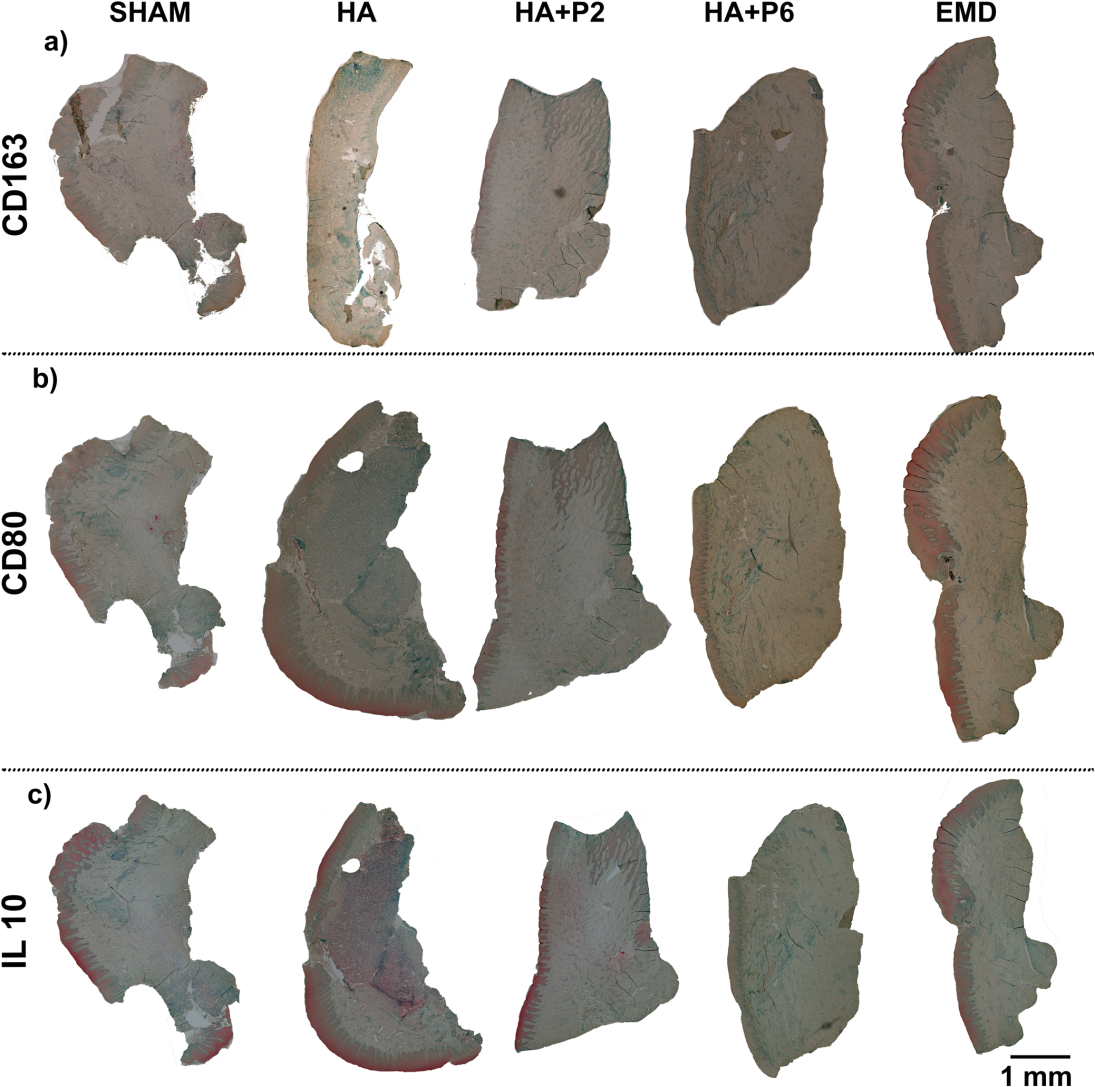
Selected representative immunohistochemistry staining of CD80 (a), CD163 (b) and IL‐10 (c).

Sham defects displayed only scattered CD163‐positive macrophages and limited CD80 immunoreactivity, with negligible IL‐10 staining, reflecting the baseline inflammatory milieu of an ungrafted defect. HA grafts elicited a pronounced accumulation of CD163‐positive cells throughout the defect, accompanied by markedly stronger CD80 labelling and a diffuse increase in IL‐10 expression, indicative of a mixed M1/M2 response to the hyaluronic acid alone (Figure 5a–c). HA + P2 reduced the CD80 signal relative to HA while maintaining moderate CD163 staining; IL‐10‐positive cells were more abundant than in the sham but less uniformly distributed than in HA + P6, suggesting a partial shift towards a proresolution phenotype. HA + P6 sections exhibited the most homogeneous CD163 labelling, minimal CD80 immunoreactivity and robust, widespread IL‐10 staining, consistent with a predominantly M2‐biased, anti‐inflammatory environment. EMD exhibited a staining pattern comparable to HA + P6, with intense signals for CD163 and IL‐10 and only faint positivity for CD80, supporting its established capacity to promote a prohealing macrophage phenotype. Collectively, the peptide‐modified HA (especially HA + P6) and EMD treatments favoured an M2‐skewed response, whereas HA alone triggered a stronger mixed inflammatory reaction (Figure 5a–c).

**FIGURE 6:**
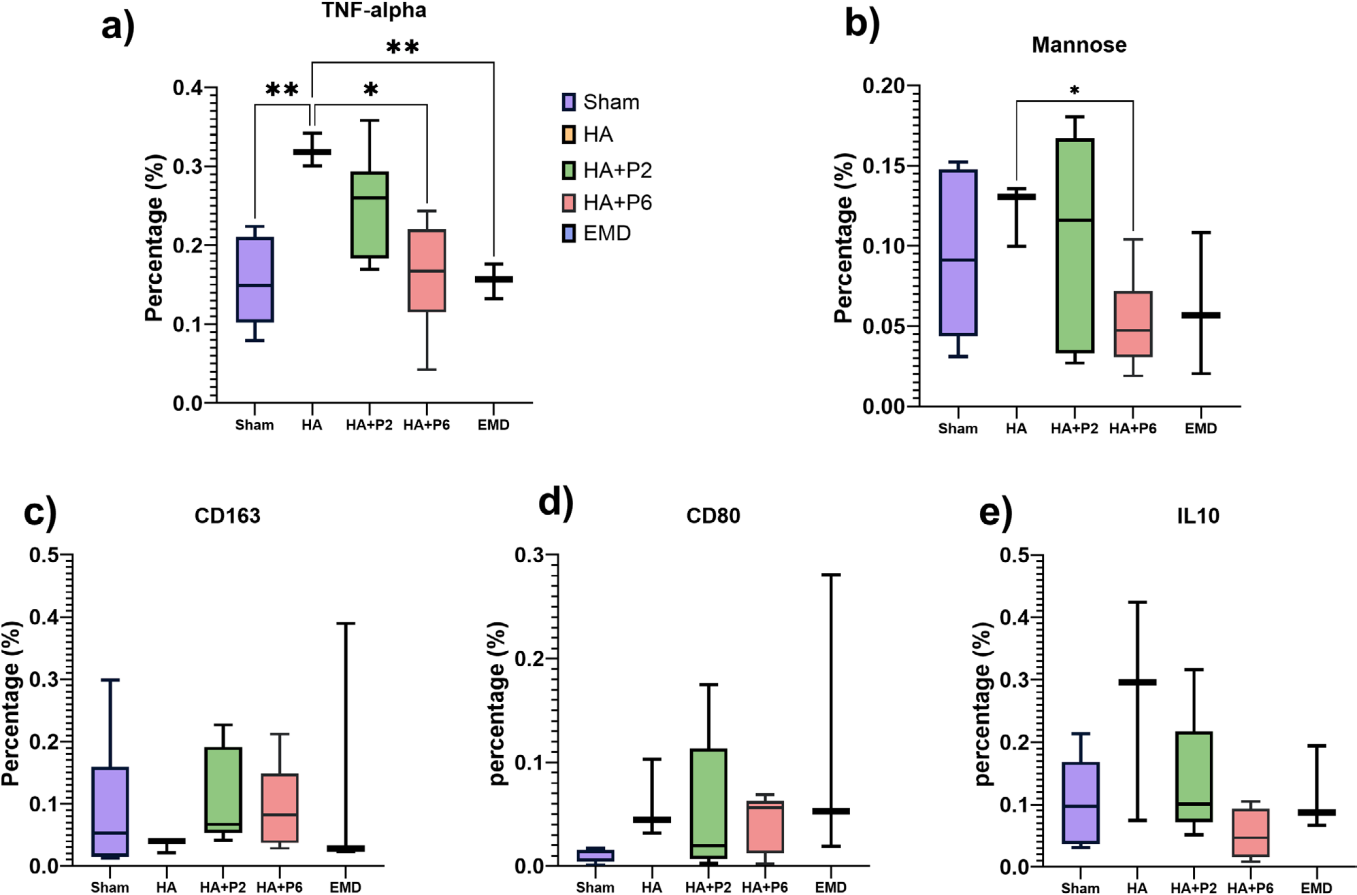
Immunohistochemical analysis of inflammatory and macrophage markers, TNF‐alpha (a), mannose (b), CD163 (c), CD80 (d), IL10 (e), across all groups (*n* = 6 defects per group, except HA and EMD with *n* = 3 defects).

Immunohistochemical analysis revealed distinct effects of treatment groups on inflammatory and macrophage‐related markers. HA treatment significantly increased TNF‐α expression compared with the Sham group (*p* = 0.0026), suggesting an elevated proinflammatory response (Figure 6a). Notably, HA + P6 significantly reduced TNF‐α levels relative to HA alone (*p* = 0.0164), indicating a potential anti‐inflammatory effect. Including other different treatments, only EMD significantly altered TNF‐α expression (Figure 6a). For CD80 and CD163, markers of pro‐ and anti‐inflammatory macrophage phenotypes, respectively, no significant differences were observed across any groups (*p* > 0.05), indicating limited modulation of macrophage polarisation under the tested conditions (Figure 6c,d). Mannose‐receptor expression was significantly increased in the HA + P6 group compared with HA (*p* = 0.0405), suggesting enhanced M2‐like macrophage activity (Figure 6b). All other comparisons were nonsignificant. Similarly, IL‐10 expression remained unchanged across all groups, with no statistically significant differences detected (*p* > 0.05), indicating that anti‐inflammatory cytokine modulation was not a dominant effect in this model (Figure 6e).

**FIGURE 7:**
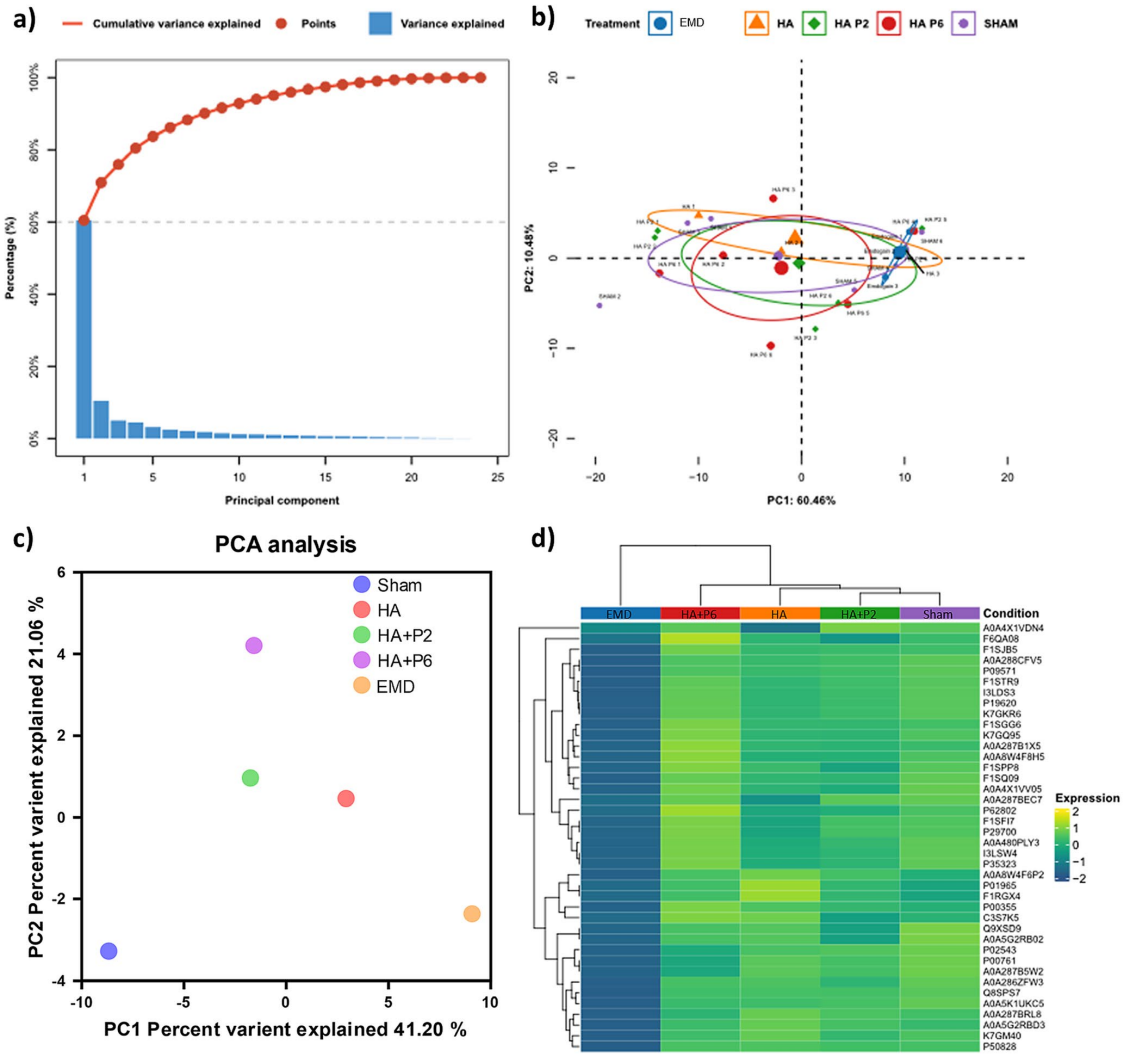
Proteomic analysis of proteins extracted from the porcine gingiva. Unsupervised machine learning clustering principal component analysis (PCA) variance plot (a). Supervised machine learning clustering partial least‐squares discriminant analysis (PLS‐DA) (b). PCA 2D scatter plot (c). Heatmap of mean expression for each group (d), (*n* = 6 defects per group, except HA and EMD with *n* = 3 defects).

Proteomics analysis revealed that EMD treatment significantly upregulated several proteins involved in wound healing, matrix organisation and cellular repair when compared to Sham and HA‐based formulations. Protein levels were normalised before proteomic analysis (Figure S1). Partial least‐squares (PLS) score plots were generated to complement the unsupervised principal component analysis (PCA) and to visualise class separation among the five treatments (Sham, HA, HA + P2, HA + P6 and EMD). The first latent variable accounted for 60% of the total variance, while the second accounted for an additional 7%, resulting in a cumulative explanatory power of 67%. Because the sum exceeded the conventional 60% threshold, the two components were sufficient to illustrate the multivariate structure (Figure 7a,b). The 95% confidence ellipses clarified how each formulation clustered in latent variable space. Although the confidence regions of adjacent groups overlapped at their margins, the centroids were displaced along orthogonal directions, indicating genuine compositional differences despite partial overlap of the ellipses. In particular, HA + P6 and EMD shifted positively along LV1, whereas HA and Sham clustered on the negative side; HA + P2 occupied an intermediate position, mirroring its mixed inflammatory profile. The relative size of each ellipse reflected within‐group dispersion rather than intergroup difference, with Sham displaying the narrowest spread and HA the widest, consistent with the histological variance reported above (Figure 7a,b). Overall, the supervised PLS results corroborated the PCA findings: peptide‐enhanced gels, especially HA + P6, drive the early tissue proteome toward the EMD‐like quadrant, whereas unmodified HA remains closer to the Sham control. The directional separation of biplot vectors therefore provides an additional layer of evidence that the proline‐rich peptide P6 confers a distinct immunomodulatory signature. The heatmap is plotted based on the top 40 proteins, as determined by the median absolute difference (MAD). The square of the distance from the data to the mean is used for the standard deviation (Figure 7c).

**FIGURE 8:**
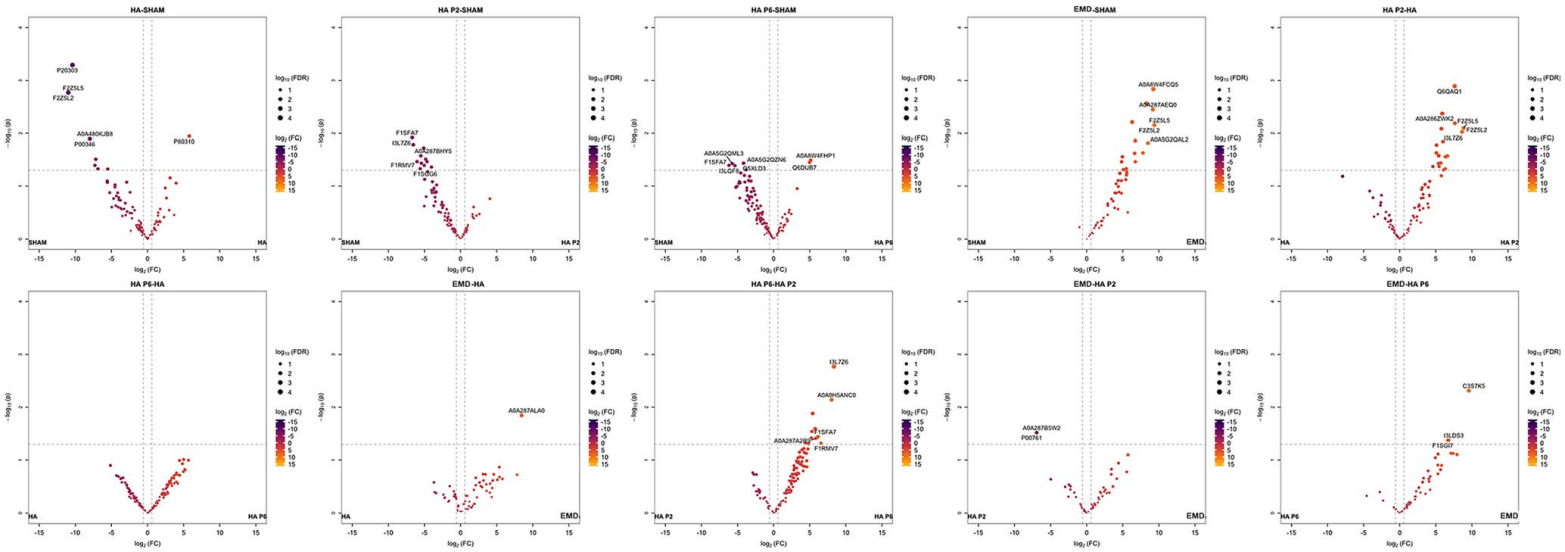
Differentially expressed protein (DEP) volcano plot comparing the protein expression level differences between the sample groups (*n* = 6 defects per group, except HA and EMD with *n* = 3 defects).

To understand the difference between the groups as shown in the heatmap (Figure 7c), one must examine the individual protein levels. Differentially expressed protein (DEP) analysis was employed to identify the differences between the groups. Figure 8 presents a series of volcano plots illustrating the differential protein expression profiles across multiple treatment comparisons, including HA, HA + P2, HA + P6 and EMD versus Sham, as well as direct comparisons between treatments. Each plot displays the log2 fold change on the *x*‐axis against the −log10 adjusted *p* value on the *y*‐axis, allowing for the visualisation of both the magnitude and statistical significance of protein expression changes. Proteins positioned to the right represent those significantly upregulated, while those on the left are downregulated in each comparison. Coloured data points indicate statistically significant proteins, with the intensity of colour reflecting the adjusted *p* value and dot size representing the magnitude of the effect. Notably, several proteins, such as Thymosin β‐10 (F2Z5L5), a histone H2B isoform (A0A287AEQ0), the keratin‐associated protein I3L7Z6 and cytoplasmic actin (Q6QAQ1), are prominently labelled due to their substantial differential expression. The plots demonstrate distinct transcriptional responses across treatments. EMD and HA + P2 comparisons reveal a strong upregulation of regenerative and remodelling‐associated proteins, whereas HA alone exhibits a more limited and predominantly downregulated profile. These patterns underscore the enhanced biological activity of peptide‐ and protein‐enriched hydrogels in modulating protein expression relevant to regeneration, particularly in the early stages of wound healing (Figure 8).

When compared to Sham, EMD significantly upregulated 19 proteins (adj. *p* < 0.16), including C3S7K5, F1SGI7, Thymosin β‐10 (F2Z5L5) and multiple histone‐related proteins (A0A8W4FCQ5, A0A287AEQ0, F2Z5L2), indicating activation of pathways associated with proliferation, ECM remodelling and cellular stress response. Compared with HA, one protein (A0A287ALA0) was moderately upregulated (logFC = 8.45), although this difference was not statistically significant after correction (adj. *p* = 0.85). Notably, compared with HA + P2, EMD downregulated trypsin‐related proteins (P00761, A0A287B5W2), suggesting a reduction in tissue remodelling activity and a shift towards a more stabilised wound environment. In the EMD versus HA + P6 comparison, several proteins, including the extracellular‐matrix protein collagen Type I α‐2 chain (C3S7K5), a keratin‐Type II cytoskeletal isoform (I3LDS3) and a 14‐3‐3–like adaptor protein (F1SGI7), were significantly upregulated (logFC > 6.6; adj. *p* < 0.56), supporting enhanced ECM activity and cellular regeneration (Table 3).

**TABLE 3:** Proteomics analysis of all up‐ and downregulated proteins only when compared to EMD. Proteins that are not significantly upregulated or downregulated (*p* value > 0.05) in the comparison are excluded from this overview.

Comparison	Protein	logFC	Ave. Expr	*t*	*p*	Adj. *p*	*B*	*x*	*y*	Significantly up‐/downregulated
EMD‐HA	A0A287ALA0	8.45	4.14	3.56	0.014	0.85	−4.59	8.45	1.85	Upregulated
EMD‐HA_P2	P00761	−6.93	2.39	−2.45	0.030	0.87	−4.59	−6.93	1.52	Downregulated
EMD‐HA_P2	A0A287B5W2	−6.93	2.39	−2.45	0.030	0.87	−4.59	−6.93	1.52	Downregulated
EMD‐HA_P6	C3S7K5	9.56	1.23	3.37	0.005	0.28	−2.64	9.56	2.31	Upregulated
EMD‐HA_P6	I3LDS3	6.77	−0.07	2.25	0.042	0.56	−3.73	6.77	1.38	Upregulated
EMD‐HA_P6	F1SGI7	6.67	−1.49	2.25	0.042	0.56	−3.73	6.67	1.38	Upregulated
EMD‐SHAM	A0A8W4FCQ5	9.24	7.23	3.91	0.001	0.07	−1.65	9.24	2.84	Upregulated
EMD‐SHAM	A0A5G2QML3	8.29	5.54	3.60	0.003	0.07	−2.01	8.29	2.56	Upregulated
EMD‐SHAM	A0A287AEQ0	9.17	6.49	3.47	0.004	0.07	−2.16	9.17	2.45	Upregulated
EMD‐SHAM	A0A8W4F6V7	6.29	5.12	3.20	0.006	0.07	−2.49	6.29	2.21	Upregulated
EMD‐SHAM	F2Z5L5	9.38	−6.25	3.13	0.007	0.07	−2.57	9.38	2.15	Upregulated
EMD‐SHAM	F2Z5L2	9.38	−6.25	3.13	0.007	0.07	−2.57	9.38	2.15	Upregulated
EMD‐SHAM	A0A4X1SK65	6.73	−4.48	2.79	0.014	0.11	−2.98	6.73	1.86	Upregulated
EMD‐SHAM	A0A5G2QAL2	8.48	0.42	2.74	0.015	0.11	−3.04	8.48	1.82	Upregulated
EMD‐SHAM	C3S7K5	7.79	1.23	2.53	0.023	0.14	−3.30	7.79	1.63	Upregulated
EMD‐SHAM	F1SGI7	6.64	−1.49	2.52	0.024	0.14	−3.31	6.64	1.62	Upregulated
EMD‐SHAM	A0A5G2R0I4	4.95	6.35	2.44	0.028	0.14	−3.40	4.95	1.56	Upregulated
EMD‐SHAM	I3L7Z6	6.74	−4.49	2.33	0.034	0.15	−3.54	6.74	1.46	Upregulated
EMD‐SHAM	Q95274	4.84	5.09	2.33	0.035	0.15	−3.54	4.84	1.46	Upregulated
EMD‐SHAM	Q29593	5.47	−3.65	2.17	0.047	0.16	−3.72	5.47	1.33	Upregulated
EMD‐SHAM	A0A8W4FPA2	5.08	1.39	2.15	0.049	0.16	−3.75	5.08	1.31	Upregulated

Proteomics profiling of the HA + P2 treatment revealed significant transcriptional changes compared to both HA alone and the Sham control, indicating distinct biological activity associated with the proline‐rich peptide. Compared with HA, HA + P2 upregulated 21 proteins (adj. *p* < 0.25), including those linked to cytoskeletal regulation (Q6QAQ1), chromatin organisation (F2Z5L5, F2Z5L2) and stress response (I3LVD5, A0A286ZWK2). Several of these proteins demonstrated high fold changes (logFC > 7), suggesting robust transcriptional activation. These data point toward an enhanced cellular remodelling and repair response following HA + P2 treatment. In contrast, when compared to Sham, HA + P2 significantly downregulated 11 proteins (adj. *p* < 0.46), including collagen I *α*‐1 (F1SFA7), keratin‐related proteins—(I3L7Z6), and iron‐uptake receptor transferrin receptor 1 (P21753), many of which are involved in structural maintenance and inflammatory regulation. Notably, the downregulation of signalling adaptors included the 14‐3‐3 ε isoform (K7GM40/YWHAE) and an uncharacterised keratin‐like protein Q95274, suggesting a shift in epithelial and immune response pathways (Table 4).

**TABLE 4:** Proteomics analysis of all up‐ and downregulated proteins only when compared to HA + P2. Proteins that are not significantly upregulated or downregulated (*p* value > 0.05) in the comparison are excluded from this overview.

Comparison	Protein	logFC	Ave. Expr	*t*	*p*	Adj. *p*	*B*	*x*	*y*	Significantly up‐/downregulated
HA_P2‐HA	Q6QAQ1	7.60	−4.46	4.13	0.001	0.14	−1.35	7.60	2.89	Upregulated
HA_P2‐HA	I3LVD5	5.89	−3.19	3.48	0.004	0.14	−2.10	5.89	2.37	Upregulated
HA_P2‐HA	A0A286ZWK2	7.64	−4.43	3.26	0.006	0.14	−2.38	7.64	2.19	Upregulated
HA_P2‐HA	F2Z5L5	8.86	−3.62	3.15	0.008	0.14	−2.51	8.86	2.10	Upregulated
HA_P2‐HA	F2Z5L2	8.86	−3.62	3.15	0.008	0.14	−2.51	8.86	2.10	Upregulated
HA_P2‐HA	Q29593	5.77	−2.33	3.13	0.008	0.14	−2.53	5.77	2.09	Upregulated
HA_P2‐HA	I3L7Z6	8.62	−3.77	3.06	0.009	0.14	−2.62	8.62	2.03	Upregulated
HA_P2‐HA	A0A286ZRU9	5.99	−1.31	2.84	0.014	0.19	−2.90	5.99	1.84	Upregulated
HA_P2‐HA	F1RX99	5.10	−2.97	2.76	0.017	0.20	−3.00	5.10	1.78	Upregulated
HA_P2‐HA	A0A4X1TY93	5.07	−2.49	2.58	0.023	0.21	−3.22	5.07	1.63	Upregulated
HA_P2‐HA	A0A5G2QZN6	5.40	−1.77	2.52	0.026	0.21	−3.31	5.40	1.58	Upregulated
HA_P2‐HA	Q5XLD3	5.40	−1.77	2.52	0.026	0.21	−3.31	5.40	1.58	Upregulated
HA_P2‐HA	A0A287A2R9	6.60	−2.37	2.51	0.027	0.21	−3.31	6.60	1.57	Upregulated
HA_P2‐HA	A0A8W4F9M4	6.32	−0.32	2.49	0.028	0.21	−3.34	6.32	1.56	Upregulated
HA_P2‐HA	F2Z5M4	5.69	−0.71	2.34	0.036	0.23	−3.52	5.69	1.44	Upregulated
HA_P2‐HA	Q9XSD9	5.39	−1.14	2.33	0.037	0.23	−3.54	5.39	1.43	Upregulated
HA_P2‐HA	A0A5G2RB02	5.39	−1.14	2.33	0.037	0.23	−3.54	5.39	1.43	Upregulated
HA_P2‐HA	F1RYZ0	5.45	−0.99	2.26	0.042	0.24	−3.62	5.45	1.37	Upregulated
HA_P2‐HA	A0A481B9A6	4.59	−2.59	2.26	0.042	0.24	−3.62	4.59	1.37	Upregulated
HA_P2‐HA	P20303	6.31	−5.32	2.20	0.047	0.25	−3.69	6.31	1.33	Upregulated
HA_P2‐HA	F1RQU2	6.08	−5.47	2.18	0.049	0.25	−3.72	6.08	1.31	Upregulated
HA_P2‐Sham	F1SFA7	−6.71	7.57	−2.80	0.012	0.46	−4.59	−6.71	1.92	Downregulated
HA_P2‐Sham	I3L7Z6	−6.55	−3.49	−2.66	0.016	0.46	−4.59	−6.55	1.79	Downregulated
HA_P2‐Sham	P21753	−5.11	5.10	−2.59	0.019	0.46	−4.59	−5.11	1.72	Downregulated
HA_P2‐Sham	A0A287BHY5	−5.52	−0.53	−2.42	0.026	0.46	−4.59	−5.52	1.58	Downregulated
HA_P2‐Sham	A0A287A2R9	−4.83	−1.44	−2.35	0.031	0.46	−4.59	−4.83	1.51	Downregulated
HA_P2‐Sham	F1RMV7	−6.04	−0.26	−2.30	0.034	0.46	−4.59	−6.04	1.47	Downregulated
HA_P2‐Sham	Q95274	−4.65	5.82	−2.30	0.034	0.46	−4.59	−4.65	1.46	Downregulated
HA_P2‐Sham	A0A8W4FCQ5	−5.44	6.35	−2.26	0.037	0.46	−4.59	−5.44	1.44	Downregulated
HA_P2‐Sham	A0A5G2QML3	−5.04	5.00	−2.22	0.040	0.46	−4.59	−5.04	1.39	Downregulated
HA_P2‐Sham	K7GM40	−4.03	5.57	−2.18	0.043	0.46	−4.59	−4.03	1.36	Downregulated
HA_P2‐Sham	F1SGG6	−5.60	0.00	−2.14	0.047	0.46	−4.59	−5.60	1.33	Downregulated

Comparative proteomics analysis revealed that HA + P6 treatment induces a distinct protein expression profile, particularly when compared to HA + P2 and Sham. In the HA + P6 versus HA + P2 comparison, 13 proteins were significantly upregulated (adj. *p* < 0.47). The most highly upregulated protein was I3L7Z6 (logFC = 8.34), followed by others involved in epithelial regulation (K7GM40, F1SFA7), immune function (Q9XSD9, P21753) and structural repair (A0A287A2R9, F1RMV7). These data suggest that HA + P6 promotes epithelial regeneration and tissue remodelling, potentially more effectively than HA + P2. Relative to Sham, HA + P6 markedly increased an uncharacterised membrane protein (A0A8W4FHP1) and keratinocyte proline‐rich protein (Q6DUB7). In contrast, it suppressed six others, notably the structural matrix component collagen Type I *α*‐1 chain (F1SFA7), the signalling adaptor 14‐3‐3 ζ/δ (Q5XLD3), and another uncharacterised protein (A0A5G2QML3). The downregulation of F1SFA7, in particular (logFC = −6.19), may indicate the attenuation of early inflammation or modulation of cytoskeletal activity (Table 5).

**TABLE 5:** Proteomics analysis of all up‐ and downregulated proteins only when compared to HA + P6. Proteins that are not significantly upregulated or downregulated (*p* value > 0.05) in the comparison are excluded from this overview.

Comparison	Protein	logFC	Ave. Expr	*t*	*p*	Adj. *p*	*B*	*x*	*y*	Significantly up‐/downregulated
HA_P6‐HA_P2	I3L7Z6	8.34	−2.54	3.65	0.002	0.22	−1.51	8.34	2.77	Upregulated
HA_P6‐HA_P2	A0A0H5ANC0	8.02	0.99	3.01	0.007	0.47	−2.48	8.02	2.14	Upregulated
HA_P6‐HA_P2	K7GM40	5.40	5.82	2.73	0.013	0.47	−2.88	5.40	1.88	Upregulated
HA_P6‐HA_P2	Q9XSD9	5.74	0.19	2.43	0.025	0.47	−3.32	5.74	1.60	Upregulated
HA_P6‐HA_P2	A0A5G2RB02	5.74	0.19	2.43	0.025	0.47	−3.32	5.74	1.60	Upregulated
HA_P6‐HA_P2	P21753	5.31	5.52	2.37	0.028	0.47	−3.40	5.31	1.55	Upregulated
HA_P6‐HA_P2	F1SFA7	6.14	8.56	2.26	0.036	0.47	−3.55	6.14	1.44	Upregulated
HA_P6‐HA_P2	A0A5K1UKC5	5.24	1.50	2.23	0.038	0.47	−3.59	5.24	1.42	Upregulated
HA_P6‐HA_P2	A0A287A2R9	5.85	−0.43	2.22	0.038	0.47	−3.60	5.85	1.42	Upregulated
HA_P6‐HA_P2	F1SFV3	4.41	5.59	2.12	0.047	0.47	−3.74	4.41	1.32	Upregulated
HA_P6‐HA_P2	F1RMV7	6.56	−0.38	2.12	0.048	0.47	−3.74	6.56	1.32	Upregulated
HA_P6‐HA_P2	Q95274	4.83	7.41	2.11	0.049	0.47	−3.75	4.83	1.31	Upregulated
HA_P6‐SHAM	A0A8W4FHP1	5.14	−2.31	2.35	0.032	0.66	−4.59	5.14	1.49	Upregulated
HA_P6‐SHAM	Q6DUB7	4.98	−2.39	2.30	0.035	0.66	−4.59	4.98	1.45	Upregulated
HA_P6‐SHAM	A0A5G2QZN6	−4.17	−0.22	−2.28	0.037	0.66	−4.59	−4.17	1.44	Downregulated
HA_P6‐SHAM	Q5XLD3	−4.17	−0.22	−2.28	0.037	0.66	−4.59	−4.17	1.44	Downregulated
HA_P6‐SHAM	A0A5G2QML3	−5.74	5.48	−2.27	0.037	0.66	−4.59	−5.74	1.43	Downregulated
HA_P6‐SHAM	F1SFA7	−6.19	8.48	−2.23	0.040	0.66	−4.59	−6.19	1.40	Downregulated
HA_P6‐SHAM	I3LQF8	−5.37	−1.46	−2.22	0.041	0.66	−4.59	−5.37	1.39	Downregulated
HA_P6‐SHAM	A0A287BFY0	−3.84	−0.67	−2.13	0.049	0.66	−4.59	−3.84	1.31	Downregulated

Proteomics profiling of the HA group relative to Sham revealed a limited but biologically relevant set of differentially expressed proteins. Only one protein, P80310, was significantly upregulated (logFC = 5.78; adj. *p* = 0.28), indicating a modest activation of protein expression related to protein transport or metabolism. In contrast, nine proteins were significantly downregulated, including an uncharacterised cytoplasmic protein (P20303), Thymosin β‐10 (F2Z5L5) and its closely related thymosin homologue (F2Z5L2), with fold changes exceeding −10 and adjusted *p* values ≤ 0.07. These proteins are associated with chromatin structure, mitochondrial function and metabolic regulation, suggesting that HA may suppress early regenerative and metabolic pathways relative to Sham. Additional downregulated proteins, such as mitochondrial oxidoreductase (A0A480KJB8) and dihydrolipoyl dehydrogenase, the E3 subunit of the pyruvate–dehydrogenase complex (P00346), also support a trend towards reduced metabolic activity (Table 6).

**TABLE 6:** Proteomics analysis of all up‐ and downregulated proteins only when compared to HA. Proteins that are not significantly upregulated or downregulated (*p* value > 0.05) in the comparison are excluded from this overview.

Comparison	Protein	logFC	Ave. Expr	*t*	*p*	Adj. *p*	*B*	*x*	*y*	Significantly up‐/downregulated
HA‐SHAM	P80310	5.78	3.29	3.03	0.011	0.28	−2.70	5.78	1.94	Upregulated
HA‐SHAM	P20303	−10.40	−5.14	−4.84	0.001	0.07	−0.73	−10.40	3.29	Downregulated
HA‐SHAM	F2Z5L5	−10.97	−4.76	−4.11	0.002	0.07	−1.46	−10.97	2.77	Downregulated
HA‐SHAM	F2Z5L2	−10.97	−4.76	−4.11	0.002	0.07	−1.46	−10.97	2.77	Downregulated
HA‐SHAM	A0A480KJB8	−7.98	−4.37	−2.97	0.013	0.28	−2.77	−7.98	1.90	Downregulated
HA‐SHAM	P00346	−7.98	−4.37	−2.97	0.013	0.28	−2.77	−7.98	1.90	Downregulated
HA‐SHAM	A0A287AEQ0	−7.17	5.98	−2.47	0.031	0.58	−3.38	−7.17	1.51	Downregulated
HA‐SHAM	F1SGG3	−7.29	4.01	−2.32	0.040	0.63	−3.57	−7.29	1.39	Downregulated
HA‐SHAM	A0A287BHY5	−6.87	−0.69	−2.23	0.047	0.63	−3.68	−6.87	1.33	Downregulated
HA‐SHAM	I3LQF8	−5.51	−1.44	−2.23	0.047	0.63	−3.68	−5.51	1.32	Downregulated

Differentially expressed protein (DEP) analysis was employed to identify the differences between the groups, with a focus on the top five upregulated and downregulated proteins (Figure 8). A detailed description is provided in the Supporting Information, and a summary is presented here. The HA + P2 group exhibited significant downregulation of proteins crucial for wound healing, including those involved in inflammation control (Alpha‐1‐antitrypsin, HSPA1A, Annexin A1, S100 calcium‐binding protein A8), cell migration (Alpha‐enolase), proliferation (Transferrin receptor protein 1) and metabolism (Alpha‐enolase), compared with the Sham group. In the HA versus Sham comparison, several proteins essential for cellular repair and immune response were downregulated, indicating suppressed wound‐healing pathways, while one protein was upregulated. The HA + P6 versus Sham analysis identified upregulated proteins potentially enhancing cellular processes (Pyruvate kinase) and downregulated proteins suggesting suppression of critical functions (Alpha‐1‐antitrypsin). EMD treatment resulted in significant upregulation of proteins associated with cell proliferation and migration (pyruvate kinase) and extracellular‐matrix formation (Histone H2B and A0A8W4F6V7), indicating enhanced wound healing compared with the Sham group. Comparisons between HA + P2 and HA revealed upregulated proteins in the HA + P2 group, including Actin and Histone H2A, suggesting an improved wound‐healing response. Additionally, EMD showed a marked upregulation of a specific protein, Brain‐abundant membrane‐attached signal protein, compared with HA, implying enhanced healing processes. At the same time, EMD downregulated the two proteins Trypsin (P00761) and trypsinogen (A0A287B5W2) compared with HA + P2, suggesting a shift towards a more stable wound environment with less remodelling (Tables 3, 4, 5).

**FIGURE 9:**
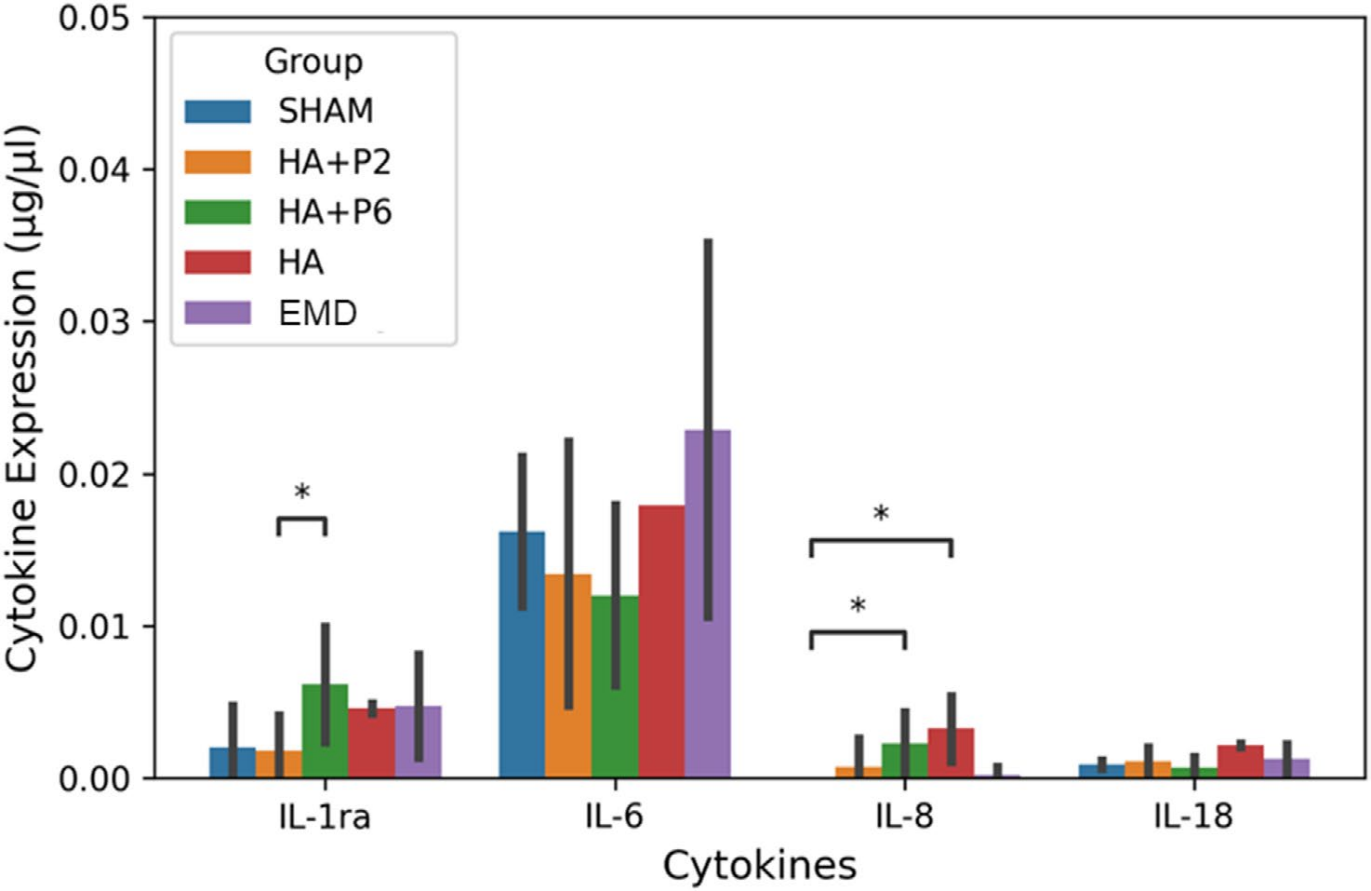
Protein expression for cytokines using a Luminex assay. *n* = 12 (6 defects) for sham, HA + P2, HA + P6 and *n* = 6 (3 defects) for HA and EMD. The data have been adjusted for sample protein concentration, and outliers have been removed.

In addition to the proteomics, Luminex was applied to determine the expression of preselected inflammatory cytokines. Most of the expression was below the detection limit of the kit, and observable levels were achieved only for IL‐1ra, IL‐6, IL‐8 and IL‐18 (Figure 9). IL‐1ra, an anti‐inflammatory cytokine, was upregulated in P6 compared to P2. Additionally, IL‐8 was upregulated in HA + P6 and HA alone compared with the sham group, which is associated with rapid wound healing (Figure 9).

**FIGURE 10:**
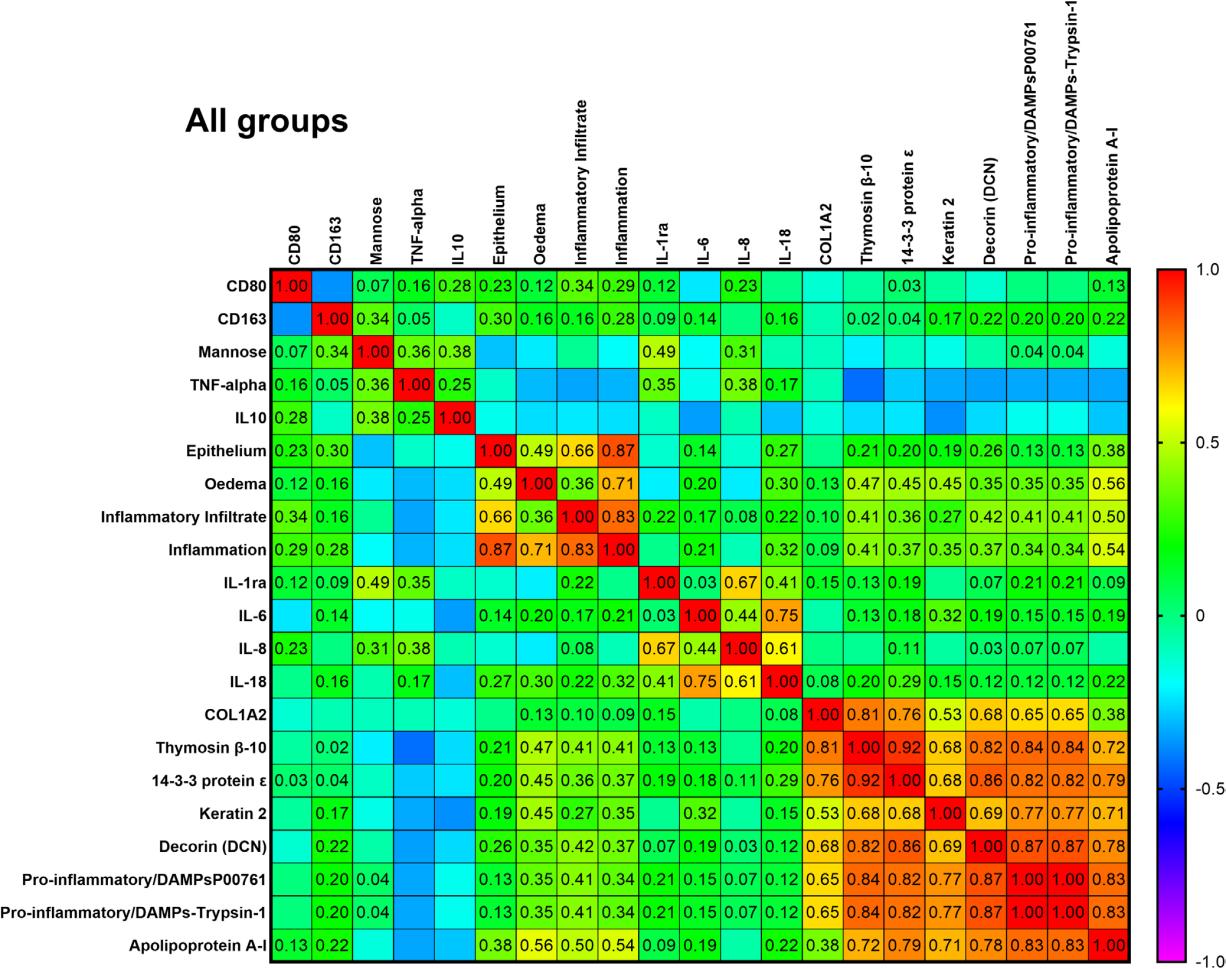
Heatmap of the Spearman correlation study between histology, IHC and key expressed proteins. The results were interpreted as follows: No correlation if |*r*| < 0.2; correlation if 0.2 < |*r*| < 0.5; and strong correlation if 0.5 < |*r*| < 1.

Spearman analysis revealed two opposing response programs that dominated the early wound milieu. A tightly interconnected inflammatory cluster—CD80, TNF‐α and the danger‐associated proteases trypsin‐1 and trypsinogen—showed strong positive intercorrelations (*ρ*≈0.47–0.52) and rose in parallel with oedema and inflammatory‐cell infiltrate, while displaying inverse relationships with epithelial tongue length. Conversely, a reparative cluster containing apolipoprotein A‐I, thymosin β‐10, 14‐3‐3 ε, keratin‐2 and decorin was positively associated with epithelial continuity and with the M2‐associated markers CD163 and mannose receptor (*ρ* = 0.72) but negatively correlated with the inflammatory proteases (*ρ* = −0.63). Oedema and the composite ‘Inflammation’ score fell between the two blocks, reflecting their mixed role in tissue damage and subsequent remodelling. Defects treated with HA + P6 or EMD aligned with the reparative axis—higher APOA1/thymosin β‐10, longer epithelium, lower CD80 and TNF‐α—whereas plain HA clustered with sham wounds inside the protease‐rich, epithelial‐poor quadrant. Thus, the correlation structure confirms that proline‐rich peptide enrichment, particularly P6, redirects the early gingival response from a TNF‐α/TNF‐α/trypsin‐centred inflammatory state towards an APOA1/keratin‐dominated reparative phenotype (Figure 10).

HA + P6 correlations are segregated into two opposing modules. A prohealing hub centred on the CD163/mannose receptor rose in parallel with epithelial extension (*ρ* = 0.93), oedema resolution (*ρ* = 0.78), and normalisation of the global inflammation score (*ρ* = 0.89). This cluster included COL1A2, decorin and the cytoskeletal triad keratin‐2, thymosin β‐10 and 14‐3‐3 ε (0.67–0.90). The counter‐module comprised TNF‐α and trypsin‐type DAMPs, mutually correlated (*ρ* = 1.00) and anticorrelated with CD163 (−0.66) and epithelial advance (−0.65); their only positive link was with CD80 (0.52). Thus, peptide enrichment insulates an M2/collagen‐remodelling program from a residual M1/TNF‐α–trypsin axis. For EMD correlation, the matrix split even more cleanly into two mirror‐image signatures. CD80, TNF‐α, and trypsin DAMPs formed a single block (*ρ* = +1) that was perfectly anticorrelated (*ρ* = −1) with an M2‐repair cluster headed by CD163, the mannose receptor, keratin‐2 and COL1A2; decorin, thymosin β‐10 and 14‐3‐3 ε tracked the latter. Oedema bridged the divide (±0.87). This near‐binary pattern indicates that EMD rapidly polarises the wound towards a CD163‐rich, collagenising, epithelialising phenotype while confining M1/TNF‐α activity to a discrete niche during the first postoperative week (Figure S3).

**FIGURE 11:**
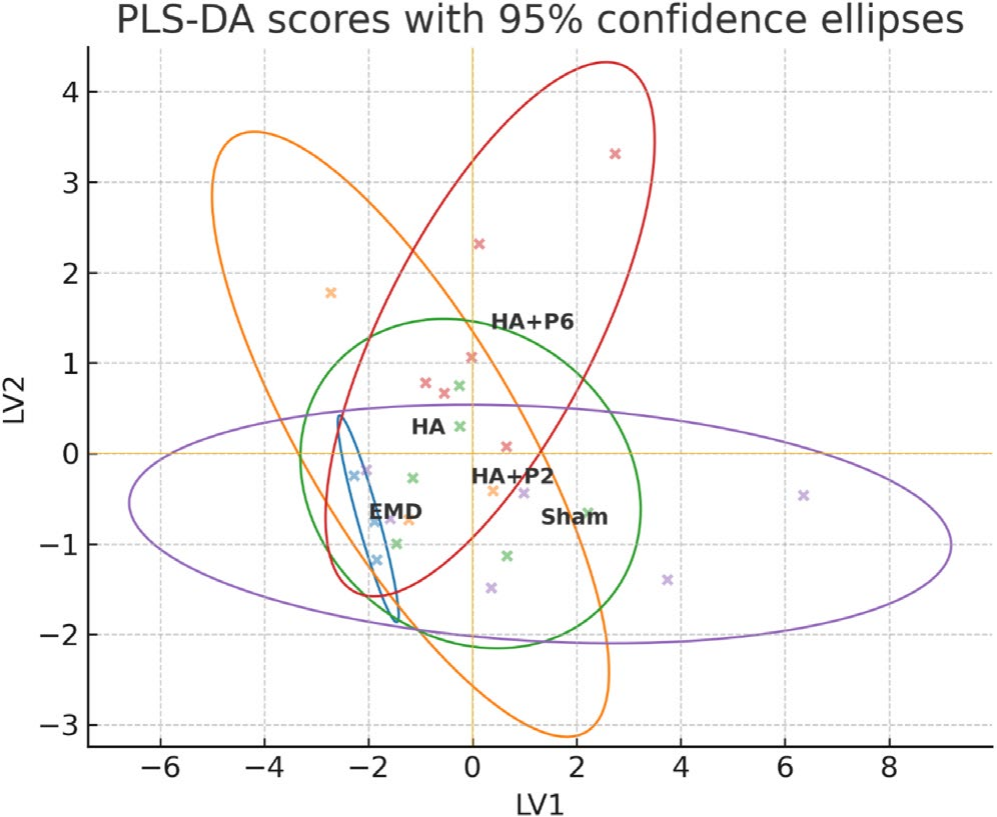
Two‐component PLS‐DA of the integrated multiomics panel. Scores of individual samples (coloured crosses) are plotted on latent variables 1 and 2; the superimposed 95% confidence ellipses outline within‐group dispersion for Sham (purple), HA (green), HA + P2 (orange), HA + P6 (red) and EMD (blue) wounds. LV1, which explains 39% of the total cross‐block variance, orders the samples along an untreated‐to‐treated gradient, while LV2 (24%) discriminates between the two peptide formulations. Absence of ellipse overlap indicates that each biomaterial induces a reproducible and statistically distinct early molecular signature.

The Spearman correlation analysis revealed a proinflammatory axis for HA + P6 and EMD (Figure S2). The reciprocal proresolution axis was driven by apolipoprotein A‐I (APOA1), Thymosin β‐10 (TMSB10), 14‐3‐3 ε (YWHAE) and Keratin‐2 (KRT2); these proteins correlated positively with epithelial integrity and CD163/mannose‐receptor staining (*ρ* = 0.46–0.71, *q* < 0.05) and negatively with the inflammatory block. To verify that a single variable set did not drive these correlations, the four data blocks were integrated using a two‐component multiblock PLS‐DA (DIABLO, with leave‐one‐pig‐out cross‐validation, achieving an overall accuracy of 87%) (Figure S3a,b). Two‐component PLS‐DA of the integrated histology, cytokine and proteomics matrix separated all five groups without centroid overlap (Figure 11). LV1 captured the main variance, ordering the samples along an untreated‐to‐treated gradient (Sham ≫ HA≈HA + P2 ≫ HA + P6 ≫ EMD), whereas LV2 resolved the two peptide formulations, positioning HA + P6 above and HA + P2 just below the origin. A permutation test (1000 random relabelling) confirmed that the between‐ and within‐cluster sum‐of‐squares ratio (4.17) was unlikely to occur by chance (*p*_perm_ = 0.047). 95% confidence ellipses were nonoverlapping, indicating tight within‐group reproducibility. Convergence of HA + P6 and EMD. Despite occupying distinct coordinates, the HA + P6 and EMD centroids lie closest in the latent space, suggesting partial convergence of their early molecular signatures. Univariate comparison, corrected for multiple testing (Benjamini–Hochberg, *q* < 0.05), identified only two significant protein differences: keratin‐associated protein I3L7Z6 (higher in EMD) and an uncharacterised keratin‐like protein Q95274 (higher in HA + P6) (Table S1). The scarcity of single‐marker disparities, combined with the multivariate proximity of the centroids, indicates that peptide enrichment shifts HA + P6 toward an EMD‐like immunomodulatory profile while retaining a discernible molecular identity. Collectively, these findings indicate that EMD induces a robust transcriptional program promoting wound stabilisation, ECM deposition, and regulated inflammation, distinguishing it from HA‐based treatments alone. HA + P6 promotes a transcriptional environment favouring epithelial remodelling and inflammation control, with a distinct regulatory pattern from both HA + P2 and Sham.

### 3.3 In Vitro Biocompatibility

Cell viability testing was used to characterise the biocompatibility of the gels (Figure S4). The HA + P6 group had significantly greater cell viability than the other groups, with a mean of 115% compared with the control, while HA had a mean of 98% and HA + P2 had a mean of 104%. All groups had cell viability according to ISO 10993‐5.

## 4 Discussion

This study aimed to determine whether the early wound‐healing response in gingival tissue could be enhanced by incorporating proline‐rich peptides in a crosslinked hyaluronic acid gel. It was done by comparison to a crosslinked hyaluronic acid gel and EMD. The focus was on interpreting signs of inflammation from various biochemical assays.

Gingival tissue has a sophisticated healing cascade. During the first 48 to 72 h after the trauma, such as a flap creation, healing is governed by the haemostatic‐inflammatory response: a fibrin clot stabilises the wound while neutrophils and then macrophages clear debris and release cytokines that set the stage for repair, changes clinicians judge by bright‐red colour, oedema and bleeding on gentle probing [31]. By about Day 4, the proliferative phase takes over; capillary‐rich granulation tissue fills the defect, and keratinocytes migrate centripetally, so epithelial coverage accelerates through the first week. A randomised clinical trial on four gingivectomy techniques found surface epithelialisation was consistently complete between Days 5 and 14, irrespective of scalpel, electrocautery or laser use [32]. A more recent prospective study that photographed standardised 3 mm buccal wounds showed 0% epithelialisation on Day 3, 61% to 69% on Day 7 and 92% to 100% by Day 14, with > 95% wound closure at the 2‐week visit [33]. The model used in this study should describe whether early clot integrity was achieved, inflammation resolved, and whether there is evidence of granulation tissue, flap stability and improved epithelial coverage [34].

Several products are in clinical use to enhance wound healing. One such product is enamel‐matrix derivative (EMD), whose primary component is amelogenin, a proline‐rich, intrinsically disordered protein [35], making it the most prominent solution. When injected into the defect site, amelogenin self‐aggregates into nanospheres, forming a temporary extracellular matrix (ECM) that facilitates tissue regeneration [35, 36]. Subsequently, the protein spheres are gradually digested by proteolytic enzymes, releasing peptides that interact with cellular receptors [37]. These peptides, among other functions, regulate the expression of dentin sialoprotein, Collagen I, BMP and TGF‐β [7, 38, 39]. Clinically, EMD has demonstrated efficacy in regenerating the periodontal ligament, alveolar bone and cementum, essential components for successful periodontal regeneration [35, 36, 37, 38, 39, 40, 41]. Recently, a hyaluronic acid gel containing 8 mg/mL HA with xylitol has entered clinical use; in a gingival‐recession trial, Pilloni et al. [40] reported that this formulation upregulated LOX mRNA, MMP‐1 protein and TIMP‐1, yet it failed to enhance angiogenesis. After instrumentation, Pilloni et al. [41] also tested a combination of hyaluronic acid and polynucleotide for treating periodontal pockets in a split‐mouth study. Throughout the 48‐week follow‐up period, both groups demonstrated clinical improvement, with no statistically significant difference between the control group (instrumentation alone) and the test group (instrumentation plus hyaluronic acid + polynucleotide). The mean PPD reduction was slightly greater in the test group (−2.08 ± 1.24 mm vs. −1.94 ± 1.19 mm), but the difference was not statistically significant; therefore, claims of superiority are unsupported. Although from the histology, an inflammatory score was obtained by averaging the grades for inflammatory infiltrate/acute infections, oedema and epithelial appearance. It was observed that there was no statistically significant difference between the groups. Contrary to what was reported in the literature [42], no anti‐inflammatory response to HA was observed in this work. The HA had a worse mean inflammatory response than the sham control. EMD had a mean like the sham but with a lower variance. At the same time, the HA with peptides showed an improved mean inflammation score, suggesting an enhanced local response. The P6 mean was slightly higher than that of P2. Interestingly, including a small quantity of the proline‐rich peptides (50 μg/mL) appears to enhance the overall response of the hyaluronic acid gel, yielding an improved inflammatory score compared to EMD. This is consistent with the in vivo results (rat oral mucosa using 37 μM EMD and P2) of Villa et al. [23], where they observed from histology that EMD had a significantly better inflammation score than the placebo on Day 1, but P2 had a significantly better inflammation score than the placebo on Days 3 and 7. Similar clinical results have also been observed for EMD. In a split‐mouth clinical trial (*n* = 12) led by Villa [43], the investigators examined the difference in cytokine levels 7 and 14 days after open flap debridement surgery, where the test group was subsequently treated with EMD. A significant difference in IL‐8 and PDGF‐BB was demonstrated after 14 days, where EMD downregulated both. In this research, the EMD group exhibited a lower IL‐8 level compared with the other groups, while IL‐6 levels were higher. It appears that IL‐6 is downregulated for HA + P6 and partially downregulated for HA + P2. Additionally, there was a comparable response among the different groups and EMD. The remaining cytokines tested were below the sensitivity limit of the kit, demonstrating one of the limitations of the Luminex method. Another major limitation of this method is that it only provides measures of preselected proteins, meaning that other up or downregulated cytokines are not detected [39], resulting in an incomplete understanding of the cascades in place.

The immunohistochemical analysis provided key insights into the inflammatory and immune‐modulatory effects of the tested treatments. HA alone significantly increased TNF‐α expression, suggesting a heightened proinflammatory response, whereas HA + P6 markedly reduced TNF‐α levels, aligning more closely with the immunological profile observed for EMD. Although no significant changes were observed in CD80 or CD163, the upregulation of the Mannose receptor in the HA + P6 group indicates a potential shift toward an M2‐like (anti‐inflammatory) macrophage phenotype. Notably, IL‐10 levels remained unchanged, suggesting that anti‐inflammatory cytokine modulation may occur through macrophage surface markers rather than soluble mediators. These findings support the immunomodulatory potential of HA + P6 and highlight its ability to attenuate inflammation, a feature critical for promoting regenerative healing in periodontal applications.

Proteomics performed 3 days after gingival detachment captured more than 650 porcine proteins and revealed that biomaterial chemistry, far more than interanimal variability, dictated the early host response. EMD produced the most prohealing signature, characterised by a broad rise in Keratin‐2, ribosomal proteins and extracellular‐matrix glycoproteins, features that align well with its clinical reputation for accelerating epithelial resurfacing and forming a provisional matrix. HA evoked the most overt inflammatory profile at the opposite end of the spectrum. In contrast, the HA + P6 shifted the molecular milieu to the proresolution pattern seen with EMD. EMD has been shown earlier to inhibit epithelial cell growth [44]. A closer look at five well‐established anti‐inflammatory ‘gatekeepers’—apolipoprotein A‐I (APOA1) [44], decorin, Thymosin β‐4, histidine‐rich glycoprotein (HRG) and 14‐3‐3 ε—highlights how subtly different surfaces manipulate innate immunity [45]. In EMD‐treated wounds, APOA1 and Thymosin β‐4 rose sharply while cationic trypsins fell by roughly seven log‐fold, indicating an early curb on PAR‐1/2 signalling and fibrin/ECM degradation. It has been demonstrated that trypsin inhibition attenuates TNF‐α and IL‐6 [46], which is consistent with the literature showing that EMD can also attenuate proinflammatory cytokines such as IL‐1β, TNF‐α and IL‐6 [47]. By contrast, HA + P2 provoked a compensatory increase in decorin, HRG and 14‐3‐3 ε when benchmarked to plain HA, yet still sat below sham levels for APOA1, Thymosin β‐4 and 14‐3‐3 ε—evidence that HA fails to resolve inflammation fully. The HA + P6 reversed this deficiency, as APOA1 and 14‐3‐3 ε rebounded, suggesting that the peptide can redirect the macrophage phenotype and restrain inflammasome activity [43, 48, 49]. The selective spike of decorin in HA + P2, but not in EMD or HA + P6, flags a potential fibrosis risk because decorin simultaneously sequesters TGF‐β and provides truncated TLR2/4 signals that appear when tissue damage is excessive [50]. Proinflammatory drivers echoed the same hierarchy. S100A8/9 and ribosomal histones, both canonical damage‐associated molecules [43, 48], were higher for HA but were decreased by EMD and HA + P6. Conversely, keratins K6/K16/K17 [51] and actin‐binding thymosin [46, 52] rose most in groups that exhibit faster epithelial restitution, again EMD and HA + P6, reinforcing the concept that inflammation resolution and epithelial migration are molecularly inseparable: APOA1, Thymosin β‐4 and 14‐3‐3 ε blunt neutrophil influx and inflammasome priming, thereby allowing keratinocytes to proliferate and migrate without protease stress. Immunohistochemistry aligned with, but also nuanced, the proteomic findings. On Day 3, none of the biomaterials had yet shifted to an IL‐10 profile. This cytokine typically peaks 4 to 5 days after wounding, consistent with the notion that the field is still undergoing an inflammatory‐to‐proliferative transition. The absence of an IL‐10 rise, therefore, does not contradict the proteomic data; instead, the early modulation of APOA1, Thymosin β‐4 and 14‐3‐3 ε by EMD and HA + P6 is expected to pave the way for a later IL‐10 surge once NF‐κB and NLRP3 priming have been dampened. TNF‐α staining, in contrast, had already diverged: Plain HA showed a significant excess relative to sham, EMD and HA + P6, while HA + P6 significantly lowered TNF‐α compared with HA. These histological observations align with the proteomics findings: The only material that failed to upregulate APOA1, Thymosin β‐4, or 14‐3‐3 ε—and that maintained high levels of S100A8/9 and trypsins—was the same material that sustained a TNF‐α surplus. Together, the data points to three design principles. First, protease shielding is pivotal; the dramatic downregulation of trypsins by EMD suggests that enamel‐matrix peptides may physically sequester or inhibit serine proteases, a property worth engineering into future coatings. Second, HA + P6 reinstates anti‐inflammatory gatekeepers and curtails TNF‐α. Third, decorin may serve as an early sentinel for fibrosis risk, rising only when an implant provokes excessive tissue stress.

The correlation structure supports a model in which two partially opposing programmes dictate early gingival healing. A proinflammatory axis—marked by mannose receptor‐positive macrophages, oedema, high TNF‐α and trypsin‐1—appears in lesions where epithelial restitution is limited. Conversely, a proresolution axis—centred on Thymosin β‐10, APOA1 and 14‐3‐3 ε—emerges when the epithelium has advanced, and TNF‐α has already subsided. Decorin's dual affiliation suggests that it is recruited early as a damage signal but then participates in matrix reorganisation once the protease burden is alleviated. Notably, the negative correlation between TNF‐α and the proresolution proteins echoes the biomaterial‐specific findings: EMD and HA + P6, which boosted Thymosin β‐10 and APOA1 in the proteomic data set, were also the only materials that prevented a TNF‐α surge histologically. In contrast, HA, which failed to raise these gatekeepers, showed the highest TNF‐α levels. Taken together, the data imply that successful biomaterials steer wounds away from a mannose^+^/TNF‐α–dominated milieu and towards an APOA1/Thymosin β‐10/14‐3‐3 ε–dominant environment, thereby enabling rapid epithelial closure and controlled collagen deposition.

### 4.1 Limitations

The model chosen was an acute gingival‐detachment model, where the defect was created by cutting into the gingiva with a scalpel to assess early wound‐healing and gingival reattachment. The model used is not suitable for assessing periodontal regeneration. Periodontal defects are created by bacteria‐induced inflammation, which means there is a trauma response to the defect creation here, as opposed to the chronic inflammation that typically occurs. The use of a scalpel breaches the mucous membrane, making the defect prone to bacterial infections. It could have been cleaned with an antiseptic agent to reduce the likelihood of infections. The small sample size made it challenging to standardise the harvest size and the embedding position. Another aspect of the model is that it focuses on the short‐term immunological response but not the complete remodelling phase; longitudinal sampling is required to link early signatures to final attachment strength [53]. Pig keratinocyte kinetics are faster than those of humans, possibly magnifying early implant effects [54]; parallel ex vivo human–gingiva explants would strengthen translational claims. A key limitation is that the gingival‐detachment model captures early soft tissue immunodynamics but does not replicate true periodontal regeneration with ligament and bone involvement. Due to the unforeseen loss of animals, two groups had only three samples, less than the power analysis recommended. However, a significant difference was still observed between the groups. EMD was not included in the CCK‐8 assay.

## 5 Conclusion

The data presented support the hypothesis that a fully synthetic, peptide‐enhanced hyaluronic acid (HA) gel can partially reproduce the early immunomodulatory signature traditionally attributed to an enamel‐matrix derivative (EMD). In the acute porcine gingival‐detachment model, the HA + P6 formulation attenuated TNF‐α and trypsin‐related danger signals, promoted CD163/mannose‐receptor expression, and upregulated proresolution proteins such as apolipoprotein A‐I, thymosin β‐10 and 14‐3‐3 ε—changes that closely paralleled those observed with EMD. Plain crosslinked HA, by contrast, maintained a TNF‐α/trypsin‐dominated profile and showed little engagement of reparative pathways.

These findings indicate that short proline‐rich peptides can endow an HA carrier with an EMD‐like, M2‐skewed early response while retaining the material's synthetic and animal‐free character. The study is limited to soft tissue healing over 6 days and does not address ligament or bone regeneration; therefore, conclusions are confined to very early immunodynamics. Longer follow‐up in true periodontal or osseous models will be required before the clinical potential of synthetic peptide‐modified HA can be fully established.

## Disclosure

AI Statement: During the preparation of this work, the authors utilised the University of Oslo (UiO) GPT and GTPZero for proofreading and readability improvement, in accordance with UiO's institutional guidelines for the use of AI. The authors reviewed and edited the content as needed and take full responsibility for the content of the published article.

## Conflicts of Interest

I.P.R. rights of the consensus peptides (US Patent US8367602B2 and connected titles) belong to Industrie Biomediche Insubri SA (Switzerland). S.P.L. was the original inventor of this patent but no longer has any financial interest in this patent. NuPep AS, which is owned solely by Ø.Ø., has a licensing right to the consensus peptides I.P.R. above. NuPep AS holds a patent for combining hyaluronic acid with proline‐rich peptides, with Mr. Øvrebø and Prof. Lyngstadaas as inventors. The other authors declare no conflicts of interest.

## Supporting information


**Appendix S1:** jre70032‐sup‐0001‐AppendixS1.docx.

## Data Availability

The data that support the findings of this study are available on request from the corresponding author. The data are not publicly available due to privacy or ethical restrictions.
