# Supplementary material for: Multiomics Comparison of Proline‐Rich Peptide‐Enhanced Hyaluronic Acid Gels Versus Conventional Regenerative Materials: An Early Wound‐Healing Model
Source: J Periodontal Res. 2025 Sep 10;60(10):1018–38. doi: 10.1111/jre.70032 (PMC12640218; doi:10.1111/jre.70032)
Supplement: Supplementary file 1 — Appendix S1: jre70032‐sup‐0001‐AppendixS1.docx. [file JRE-60-1018-s001.docx]

**Multi-Omics Comparison of Proline-Rich Peptide-Enhanced Hyaluronic Acid Gels versus Conventional Regenerative Materials: An Early Wound-Healing Model**

Øvrebø Ø et al.

**Supplementary information:**

**Section 1. Protein expression analysis and viability analysis**


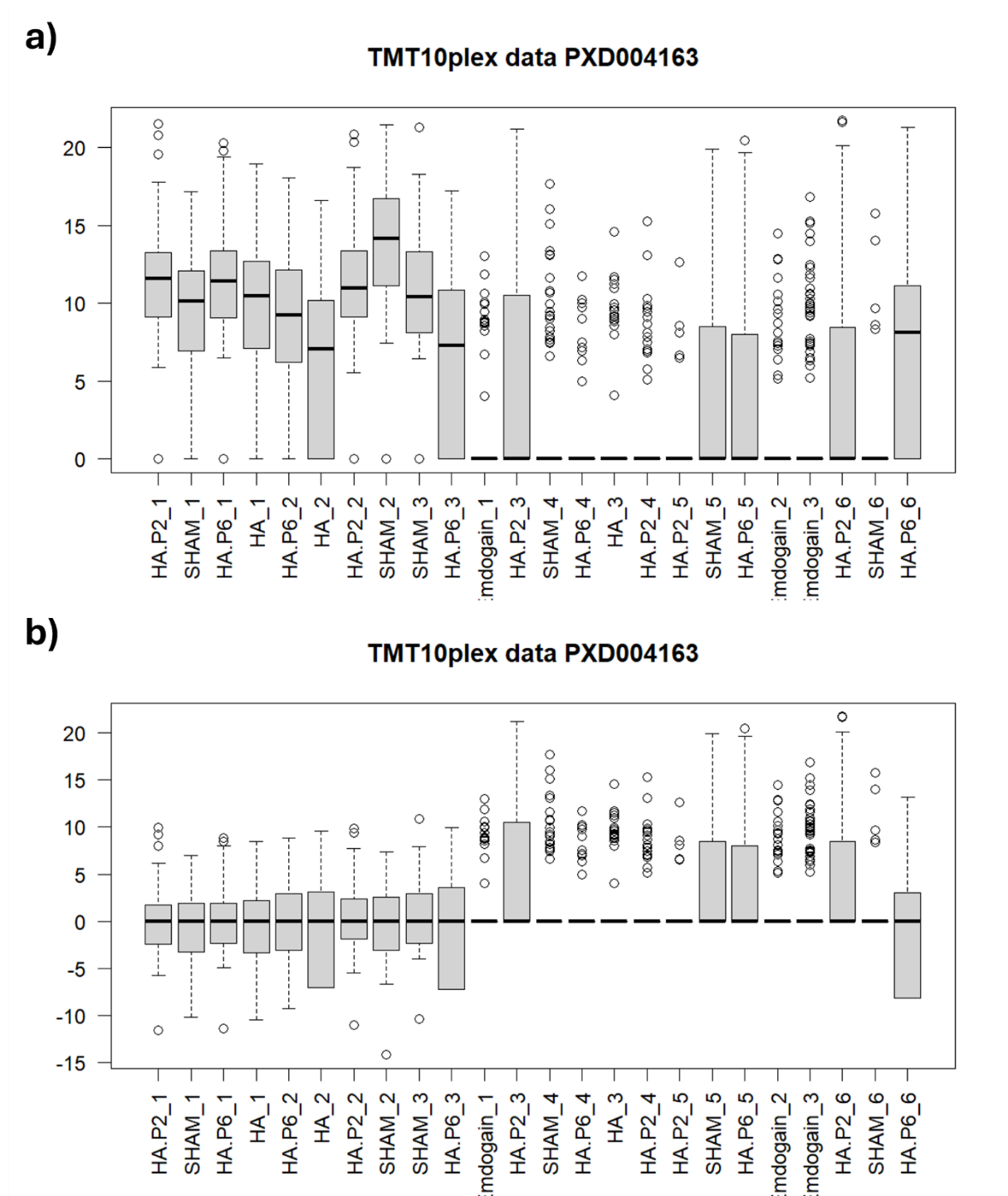


**Figure S1:** Protein levels before (a) and after (b) equal median normalisation from proteomic analysis

**
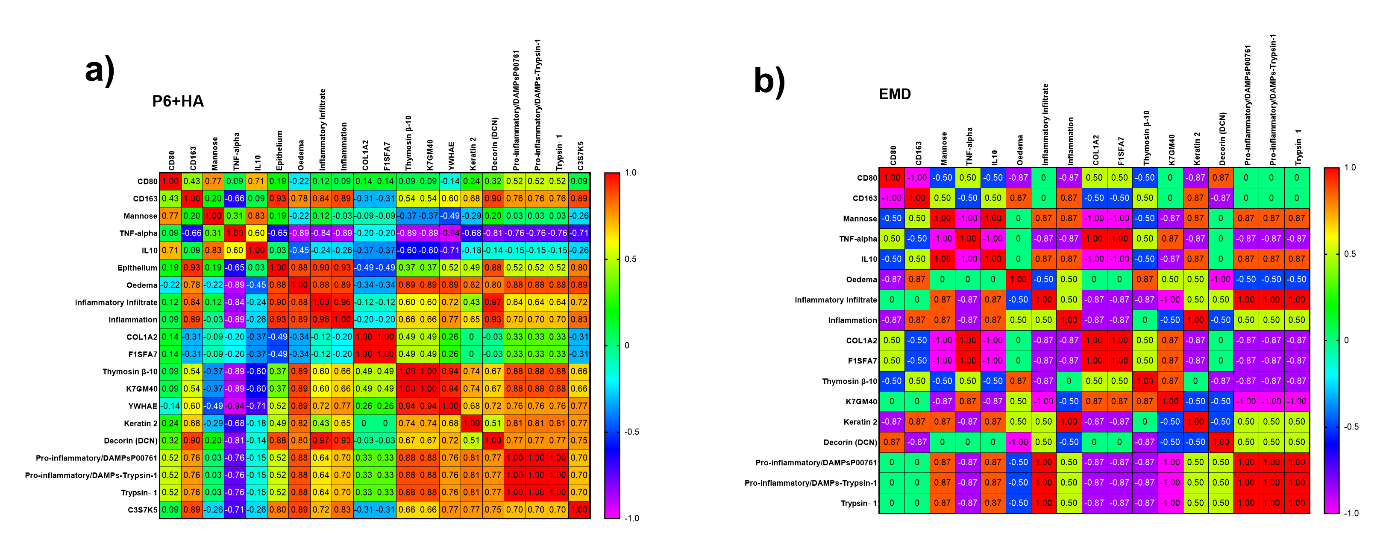
**

**Figure S2:** Heatmap of the Spearman correlation study between key proteins only for HA+P6 and EMD. The results were interpreted as follows: no correlation if |r| < 0.2; correlation if 0.2 < |r| < 0.5; and strong correlation if 0.5 < |r| < 1.


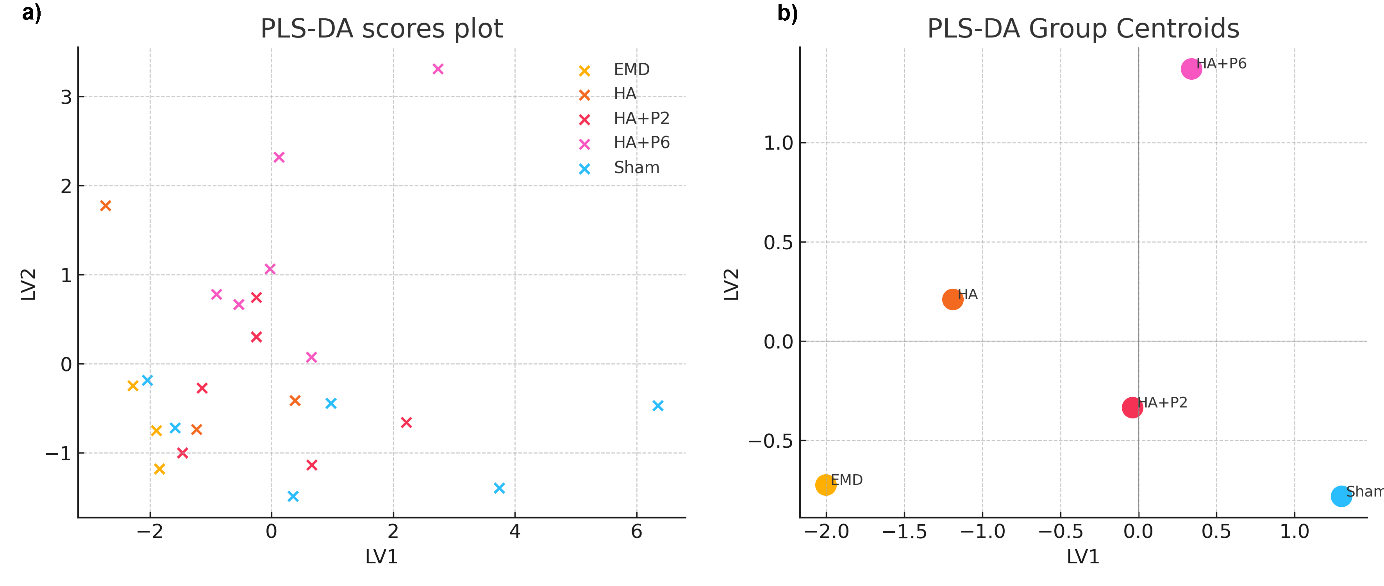


**Figure S3:** Two-component PLS-DA of the early multi-omics response. (a) Scores plot of individual wounds on latent variables 1 and 2; colours denote the five treatment groups (EMD, HA, HA + P2, HA + P6, Sham). Each symbol represents one quartile defect (n = 6 per group, except for HA and EMD, which have n = 3). (b) Group centroids (means ± 0 along both axes) extracted from the same score matrix. Centroids illustrate a treatment gradient along LV1 (Sham → HA → EMD) and highlight the distinct positioning of the two peptide formulations on LV2, with HA + P6 showing the most considerable positive shift.

| Protein | Adj_p |
| --- | --- |
| CD80 | 0.03 |
| CD163 | 0.03 |
| Mannose | 0.03 |
| TNF-alpha | 0.03 |

**Table S1**: Univariate HA + P6 vs EMD comparison, After Benjamini–Hochberg correction (q < 0.05) only two proteins differed significantly between HA + P6 and EMD


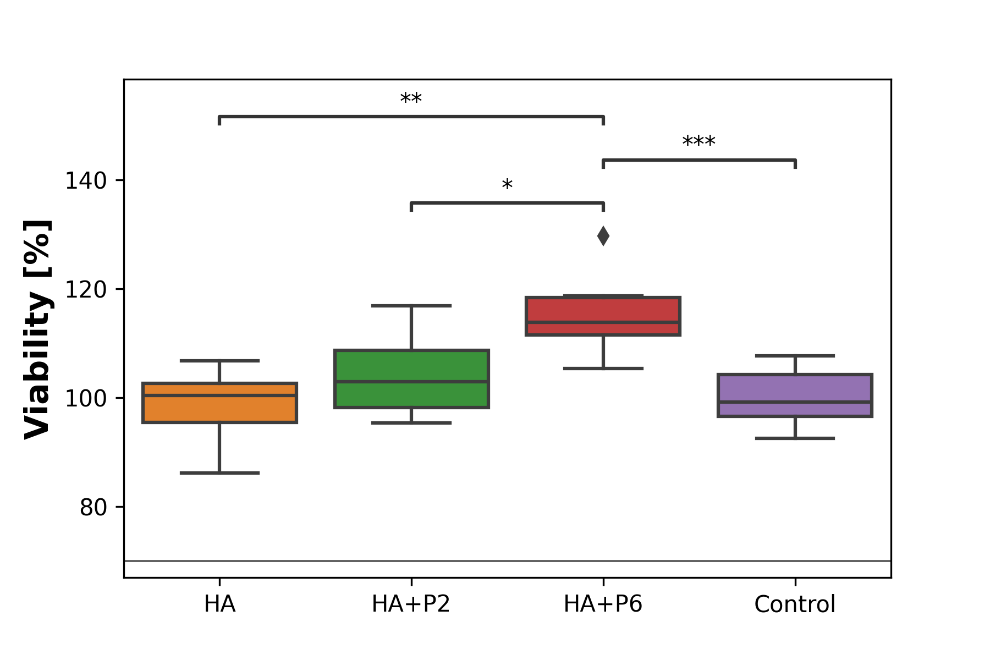


**Figure S4:** Cell viability was measured using CCK8 assay (right). n=8, *p≤0.5, **p≤0.01, ***p≤0.001.

**Section 2. Histology grading and images**

**Supplementary Table S2:**

Grading of histology samples. Grading system: 1 (compromised), 2 (somewhat compromised), 3 (normal physiology). LL: Lower Left, LR: Lower Right, UL: Upper left: UR: Upper Right.

| **Group** | **Pig Position** | **Epithelium** | **Oedema** | **Inflammatory Infiltrate** | **Tissue Morphology Score** | **Comments** |
| --- | --- | --- | --- | --- | --- | --- |
| HA+P2 | 1_LL | 3 | 2 | 3 | **2.67** | Chunk of inflammatory infiltrate the bottom and top, but not within the tissue. Some coagulum with some inflammatory infiltrate around suggesting remodelling. Some oedema in the centre region |
| Sham | 1_LR | 3 | 1 | 2 | **2.00** | Good epithelium, but some inflammatory infiltrate and a fair bit of oedema |
| HA+P6 | 1_UL | 2 | 2 | 1 | **1.67** | Epithelium is broken in what seems to have been a tissue fold. Large amounts of inflammatory infiltrate. Some oedema by infiltrate |
| HA | 1_UR | 1 | 1 | 1 | **1.00** | Weakened and irregular epithelium breached by what seems to be an active infection. Intense inflammation with much infiltrate. Much oedema around infiltrate in bottom region. coagulum in the top region, with thick fibrous tissue around, but no inflammatory cells suggesting ongoing remodelling. |
| HA+P6 | 2_LL | 3 | 2 | 3 | **2.67** | Cut wrong, which is why epithelial is only in the bottom. The bottom part is strong and keratinised. Some oedema. |
| HA | 2_LR | 2 | 1 | 2 | **1.67** | Some thinning of epithelium. Sequestration of necrotic cells, suggesting inflammation. A lot of oedema |
| HA+P2 | 2_UL | 3 | 3 | 3 | **3.00** | Strong epithelium with keratinisation throughout. Possibly some indications of failed reattachment due to epithelium on three sides, but can be artefact due to cutting direction |
| Sham | 2_UR | 1 | 1 | 1 | **1.00** | Severe inflammation with infiltrate. A lot of oedema, no epithelium. |
| Sham | 3_LL | 2 | 2 | 2 | **2.00** | Some irregularities to epithelium, but still strong and keratinised. Some oedema, and some infiltrate spread out throughout. The bottom left region is believed to be muscle fibres and the cavity to have been part of the mandibular nerve canal |
| HA+P6 | 3_LR | 2 | 2 | 2 | **2.00** | Some weakening of epithelium, some oedema throughout, and some regions with a lot of infiltrate. Some coagulum, but no infiltrate around that would’ve suggested ongoing remodelling. |
| HA+EMD | 3_UL | 2 | 2 | 1 | **1.67** | Thinning of epithelium. Some oedema. A lot of infiltrate around and sharpening of rete ridges suggesting inflammation. |
| HA+P2 | 3_UR | 2 |  | 2 | **2.00** | Some thinning of epithelium, some infiltrate, and signs of pus at the outer parts. The cutting direction makes it hard to assess oedema, hence it was omitted in this case. |
| Sham | 4_LL | 1 | 2 | 1 | **1.33** | Thinning of epithelium, breached over a region. A fair bit of infiltrate and indication of pus exiting the tissue. Some oedema |
| HA+P6 | 4_LR | 2 | 1 | 1 | **1.33** | Some epithelium thinning. A lot of oedema throughout and infiltrate (particularly left side). |
| HA | 4_UL | 2 | 1 | 1 | **1.33** | Epithelium most intact with keratinisation, but with thinning throughout and breached in a region. A lot of oedema and a lot of infiltrate. Signs of an active infection in the top. |
| HA+P2 | 4_UR | 3 | 2 | 1 | **2.00** | Strong epithelium with keratinisation. Breached one place, likely due to an infection in an abscess Sign of necrotic tissue and infection with pus deep into the tissue. Some oedema. |
| HA+P2 | 5_LL | 1 | 1 | 1 | **1.00** | Epithelium only in a small region, but very thin appearance. Inflammatory infiltrate throughout. A lot of oedema as well. Abscess in middle with necrotic tissue. |
| Sham | 5_LR | 2 | 1 | 2 | **1.67** | Epithelium in most regions, but irregular and a fair bit of thinning. Large gap in the middle believed to be oedema related. Signs of sequestering in the top region with necrotic cells. |
| HA+P6 | 5_UL | 3 | 3 | 3 | **3.00** | Strong epithelium with keratinisation throughout. Epithelium in . No signs of oedema, nor infiltrates. |
| HA+EMD | 5_UR | 2 | 1 | 2 | **1.67** | Wrinkled epithelium, a lot of oedema, some inflammatory infiltrate close to the oedema. |
| HA+EMD | 6_LL | 2 | 2 | 2 | **2.00** | Epithelium breached with a necrotic wound, otherwise strong with keratinisation. Presence of oedema and inflammatory infiltrate. |
| HA+P2 | 6_LR | 2 | 2 | 1 | **1.67** | Hypoplastic epithelium, but mostly strong with keratinisation. Some oedema. Some infiltration and sharpening of rete ridges, suggesting inflammation. |
| Sham | 6_UL | 2 | 2 | 1 | **1.67** | Hypoplastic epithelium, some oedema, a lot of inflammatory infiltrate. |
| HA+P6 | 6_UR | 3 | 2 | 2 | **2.33** | Strong epithelium with keratinisation, although minor thinning. Some oedema. Local abscess. Some infiltration around, particularly top right corner. Some coagulum with some inflammatory cells around for remodelling. |

**Images pigs – LHS: images of intervention area moments after euthanasia. RHS: corresponding histology images with Masson Goldner Trichrome staining. Scale bars = 1 mm.**

**Supplementary Figure S5 - Pig 1:**

**
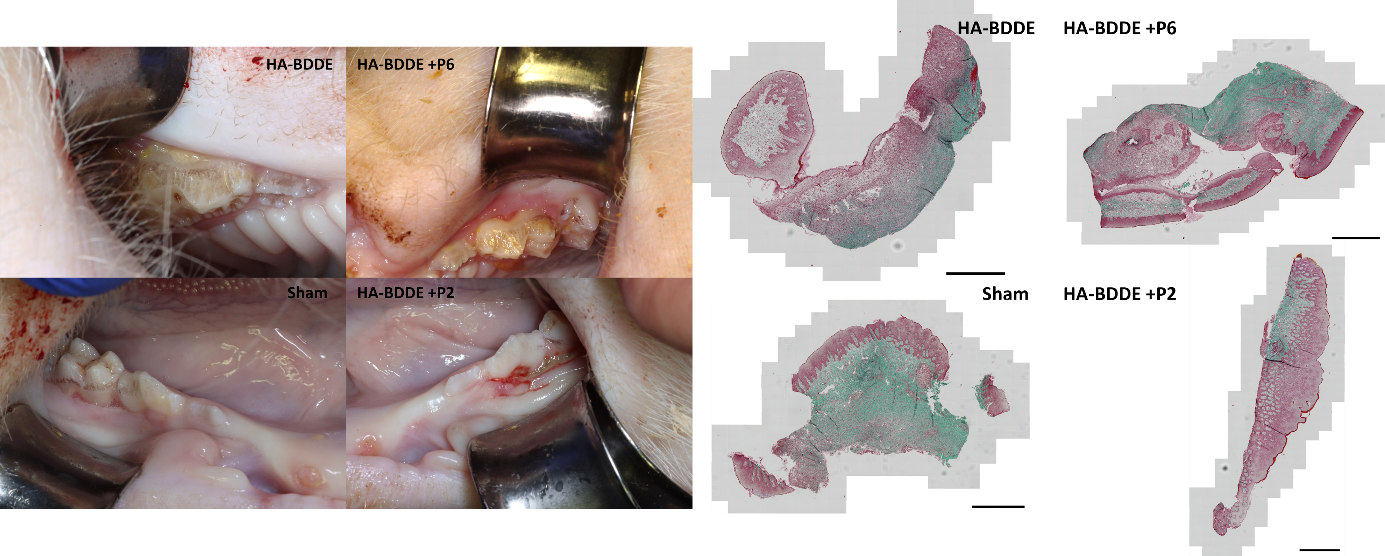
**

**Supplementary Figure S6 - Pig 2:**

**
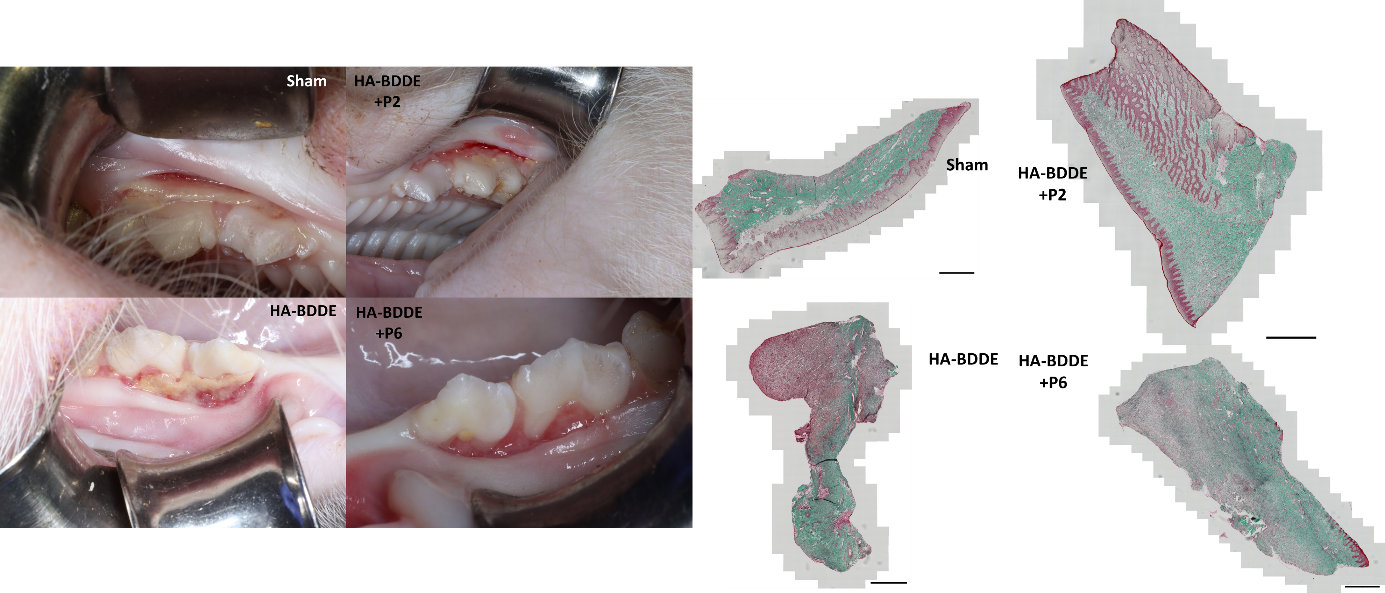
**

**Supplementary Figure S7 - Pig 3:**

**
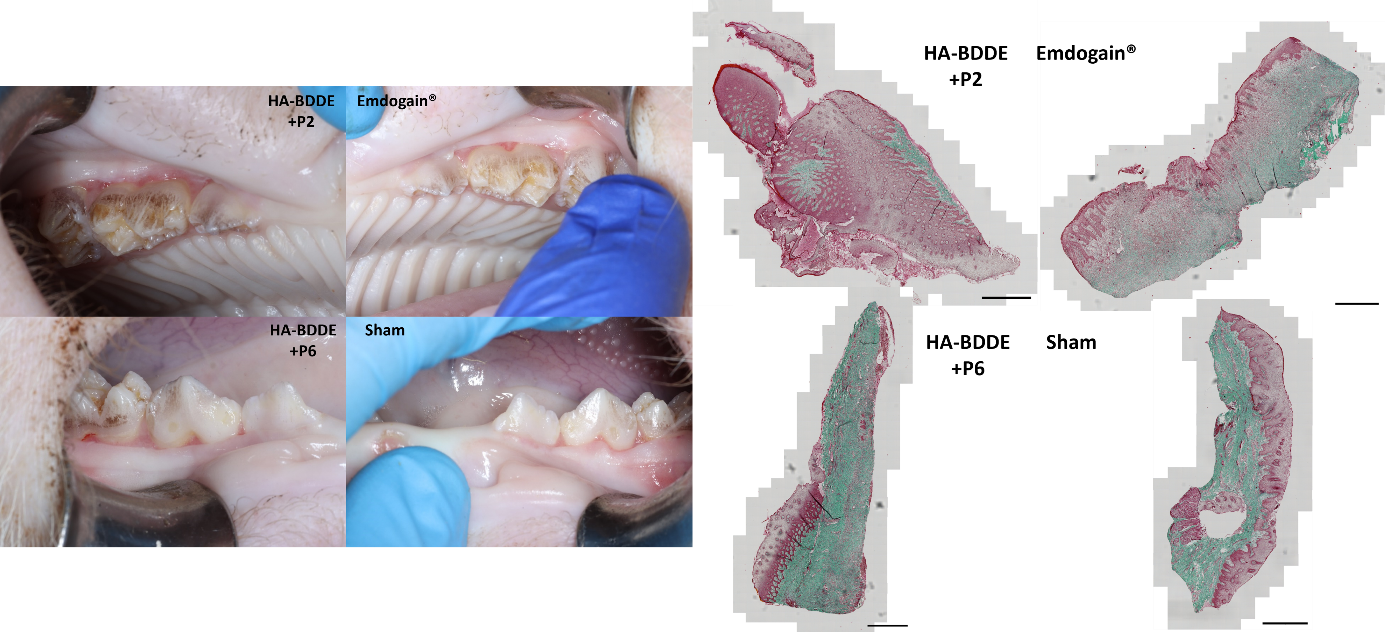
**

**Supplementary Figure S8 - Pig 4:**

**
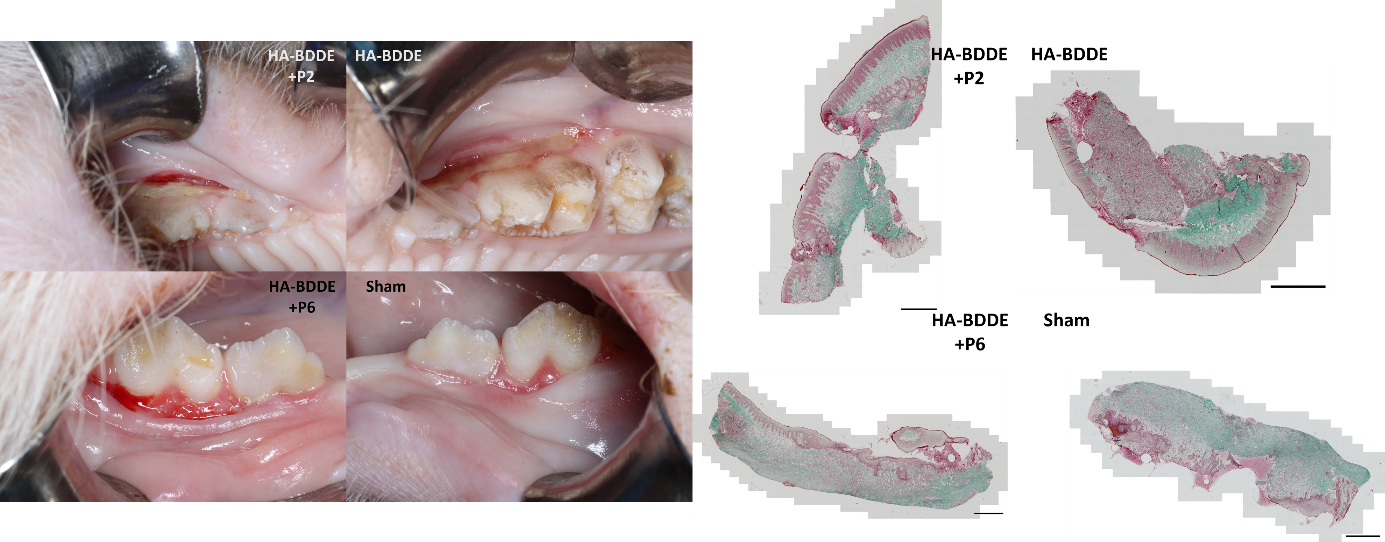
**

**Supplementary Figure S9 - Pig 5:**

**
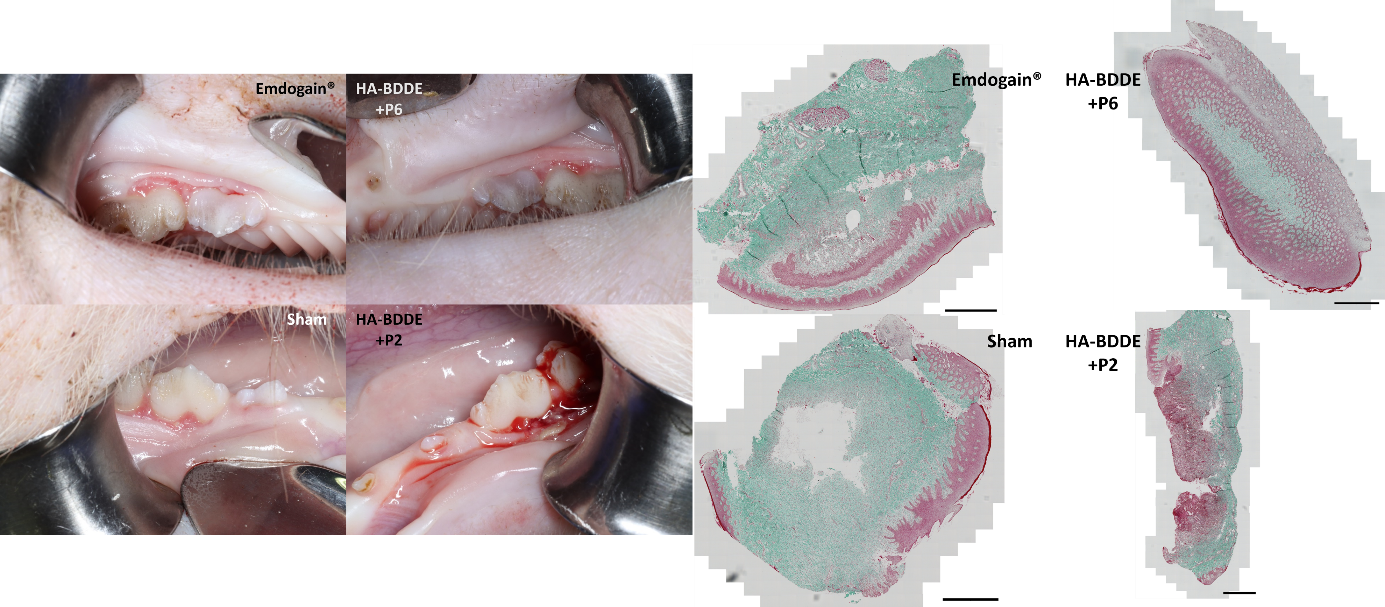
**

**Supplementary Figure S10 - Pig 6:**

**
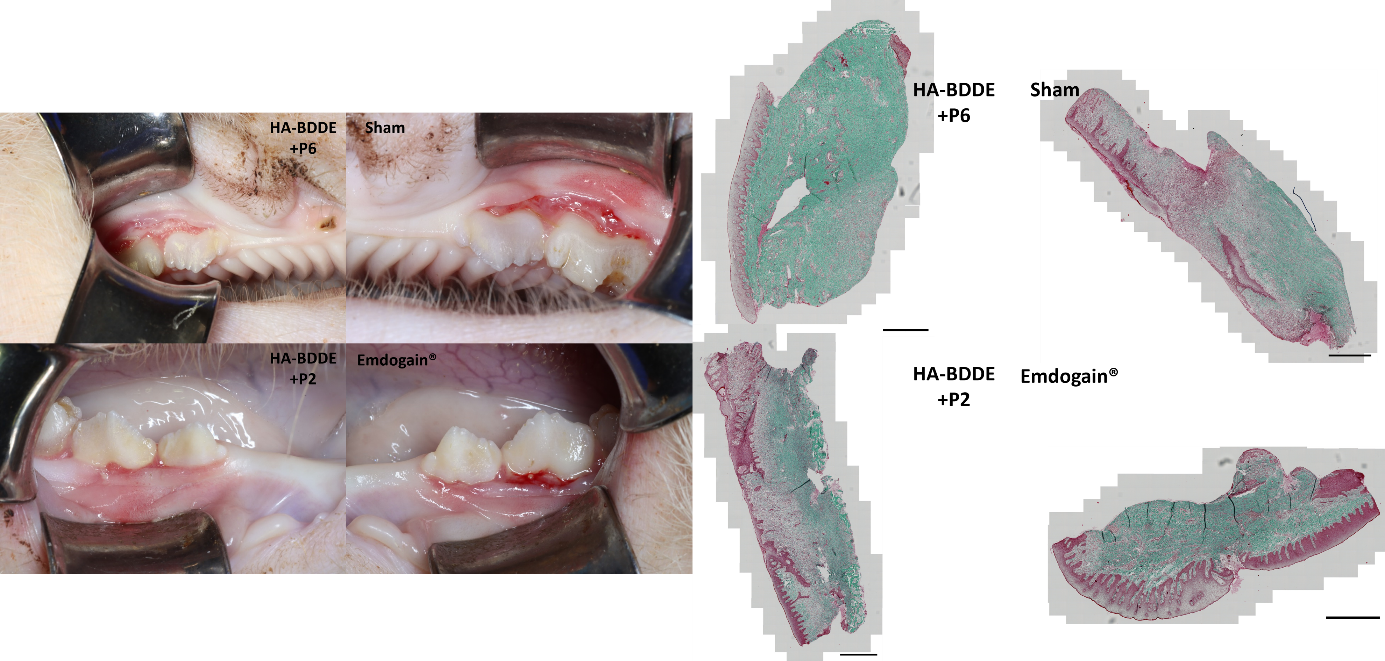
**

**Images pigs – Toluidine blue stains. This stain was prone to artefacts (purple needle-like spots. Scale bars = 1 mm.**

**Supplementary Figure S11 - Pig 1:**

**
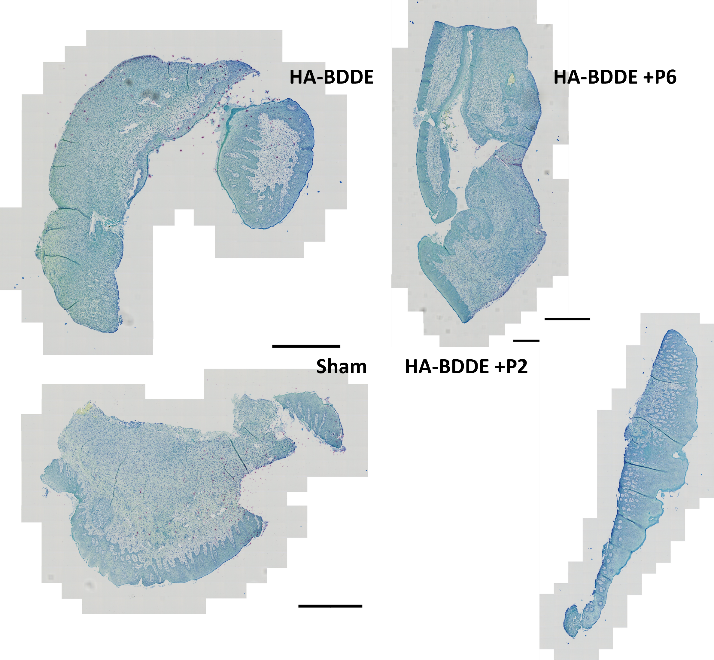
**

**Supplementary Figure S12- Pig 2:**

**
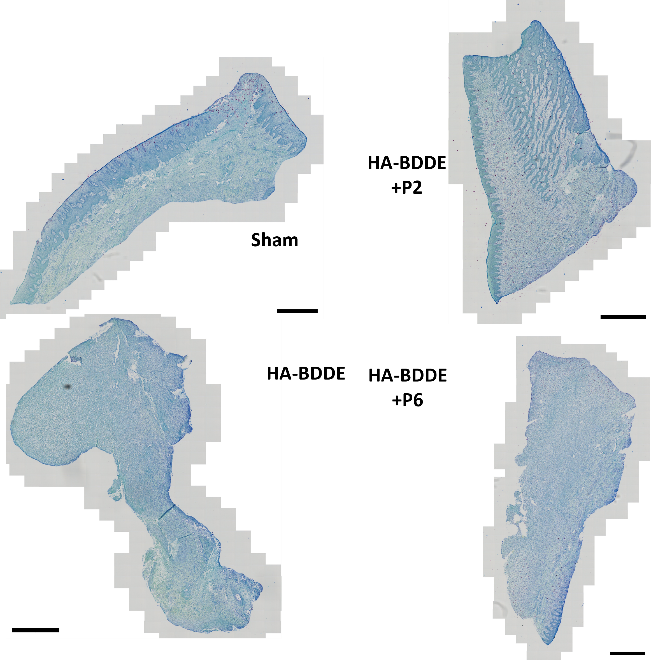
**

**Supplementary Figure S13 - Pig 3:**

**
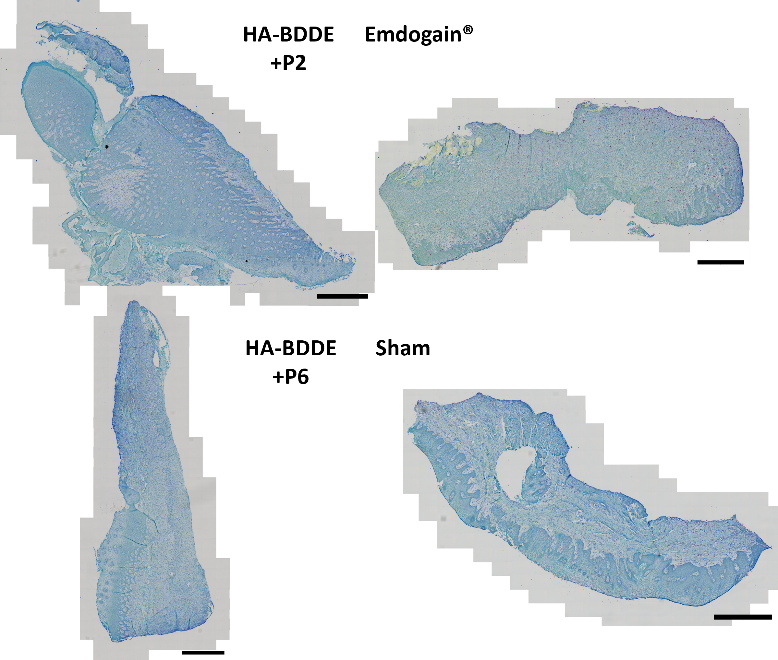
**

**Supplementary Figure 14 - Pig 4:**

**
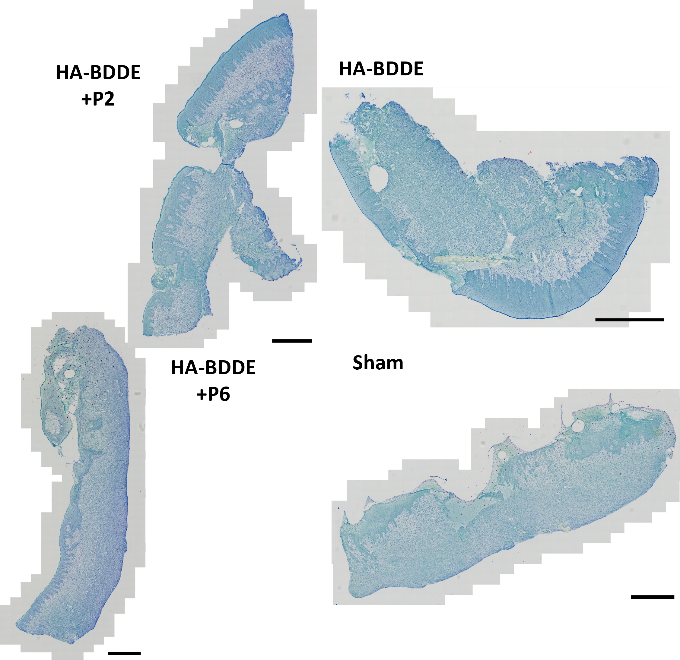
**

**Supplementary Figure S15 - Pig 5:**

**
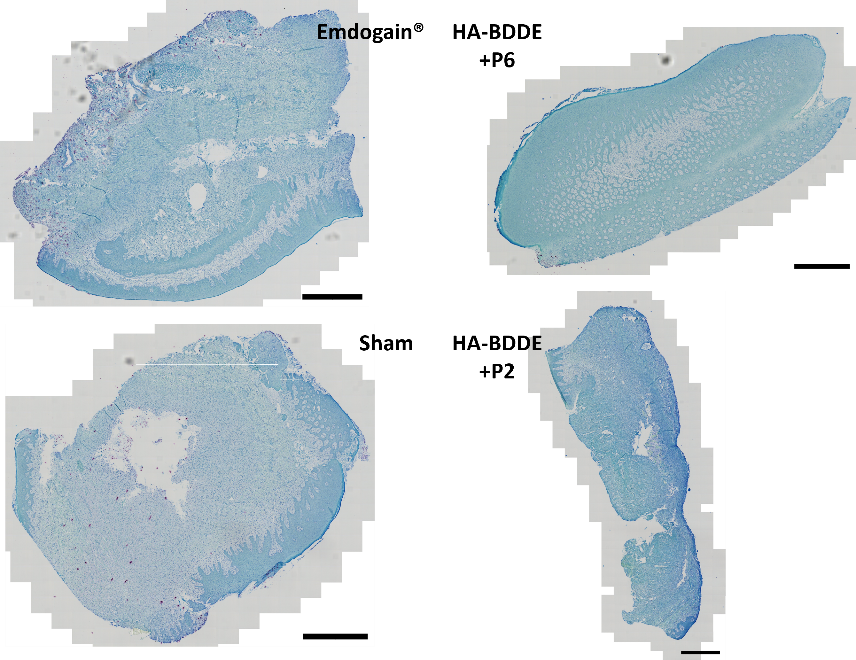
**

**Supplementary Figure 16 - Pig 6:**

**
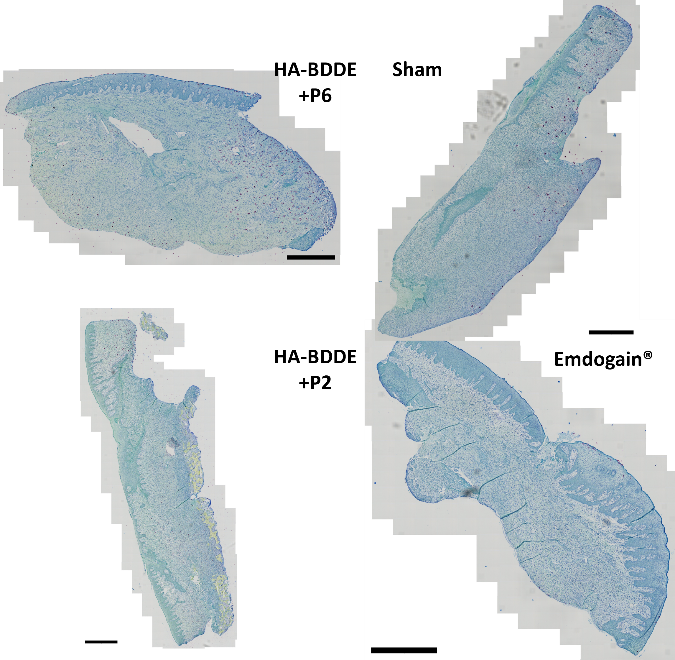
**
